# Unveiling Adenine H‑bonded Hexads: Hierarchical Self-Assembly for Helical Columnar Functional Materials

**DOI:** 10.1021/jacsau.5c00256

**Published:** 2025-06-19

**Authors:** Alejandro Martínez-Bueno, Génesis M. Valencia-Vásconez, Roberto Termine, Attilio Golemme, Josu Ortega, César L. Folcia, José M. Granadino-Roldán, Amparo Navarro, Raquel Giménez, Teresa Sierra

**Affiliations:** † Instituto de Nanociencia y Materiales de Aragón (INMA), CSIC-Universidad de Zaragoza, 50009 Zaragoza, Spain; ‡ Departamento de Química Orgánica, Facultad de Ciencias, Universidad de Zaragoza, 50009 Zaragoza, Spain; § CNR-NANOTEC SS di Rende, Dipartimento di Fisica, Università della Calabria, 87036 Rende, Italy; ∥ Department of Physics, Faculty of Science and Technology, UPV/EHU, 48940 Bilbao, Spain; ⊥ Departamento de Química Física y Analítica, Facultad de Ciencias Experimentales, Universidad de Jaén, Campus Las Lagunillas, 23071 Jaén, Spain

**Keywords:** adenine, hydrogen bonding, rosette, supramolecular chemistry, liquid crystal, chirality, organic semiconductor

## Abstract

This manuscript reports on an unusual self-assembly of small adenine-based molecules leading to complex, functional systems. Molecules feature an adenine nucleobase substituted at the N9 position with a triarylamine unit through a flexible spacer. Hydrogen bonding interactions prompt the formation of unprecedented adenine hexameric rosettes, which organize in dimers and then into helical columnar assemblies exhibiting hexagonal columnar liquid crystal phases, even with nonchiral molecules. Theoretical calculations including geometry optimization and prediction of vibrational modes have provided essential insight into the configuration of hydrogen bonds between adenine units to form stable hexads, and experimental and simulated X-ray diffraction (XRD) patterns are consistent with the unique helical self-assembly. Furthermore, molecular design including chirality in the flexible spacer and triarylamine electron-donor units steers these nanostructured materials toward functionalities related to the control of supramolecular chirality and semiconductivity. This is confirmed by thin film circular dichroism measurements for chirality and the space charge-limited current method for hole transport.

## Graphical abstract

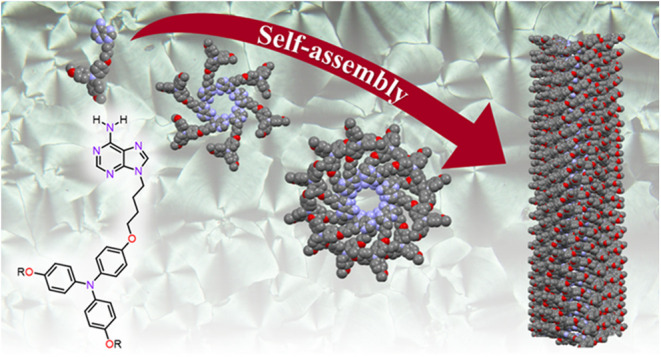

## Introduction

Intermolecular interactions in soft, self-organized functional materials produce distinctive structural characteristics, and the generation of first-hand information about these assemblies paves the way for developing complex architectures with alluring technological properties.^1–4^ For this purpose, supramolecular chemistry renders essential concepts for endowing low-molecular-weight molecules with self-organizing capabilities.^5,6^ In this context, liquid crystals (LCs) exemplify self-organized materials in which hydrogen (H)–bonds, as dynamic and directional noncovalent interactions, in combination with π-stacking may work as a powerful tool to construct functional nanostructures through hierarchical self-assembly.^7,8^ Other factors, such as the shape complementarity can also influence the formation of complex supramolecular LC architectures.^9^

A precise molecular design is fundamental to guide the organization of the molecules, and biomolecules constitute a valuable source of inspiration in this respect. Thus, artificial systems have been developed using H-bond DNA/RNA base pairing.^10–13^ Apart from that, some nucleobases are able to form macrocycles through self-complementary H-bonds.^14^ A notable example is the guanine (G)-quartet motif,^15^ which has been reported to stack in one-dimensional (1D) nanostructures in the presence of cations, leading to the formation of 1D soft nanostructures,^16,17^ lyotropic^18,19^ and thermotropic^20^ LC behavior and, using guanine-oligothiophene conjugates, enabling charge transport properties within columnar (Col_h_) phases.^21^

Less commonly studied is the formation of adenine macrocycles, with tetrads seldom described in some DNA or RNA oligonucleotides.^22^ Since adenine is an asymmetric molecule, it can form three different types of tetramers depending on which nitrogen atom in the aromatic ring engages in H-bonding with the amino group.^23,24^ Of the three tetramers predicted, only two have been experimentally observed.^25,26^ This variability evidence the complexity for understanding adenine homo-assemblies in the quest to functional architectures.

Here, we report on this fact by describing a new supramolecular homoassembly of adenine using three small molecule derivatives, **Aden-C**_**4**_ **TPA**, **Aden-(** ***R*** **)­C**_**3**_ *TPA, and **Aden-(** ***S*** **)­C**_**3**_ *TPA. Each molecule features a triarylamine (TPA) electron-donor unit attached at the N9 position of the adenine via an aliphatic spacer (Figure 1a). Remarkably, these compounds gave rise to unprecedented hierarchical assemblies in bulk. Unveiling the complex architectures was possible by a combination of experimental and theoretical studies. H-bonding interactions prompt the formation of hexads in the form of hexameric rosettes, which organize in dimers, and then into helical columnar assemblies exhibiting hexagonal columnar LC phases, even with nonchiral molecules. (Figure 1b). To the best of our knowledge, these compounds represent the first report of cyclic hexads formed from adenine units and, in addition, the only example forming columnar mesophases.^27^ Furthermore, molecular features such as chirality and TPA play a crucial role in the assembled nanostructure, endowing the materials with supramolecular chirality and hole transport properties while offering more synthetically straightforward alternatives to complex helical assemblies^1^ and TPA-based semiconductors.^28,29^ Hence, the new molecules reported serve as a new platform to develop new functional systems out of small molecule designs consisting of adenine/flexible spacer/functional unit.

**Figure 1:**
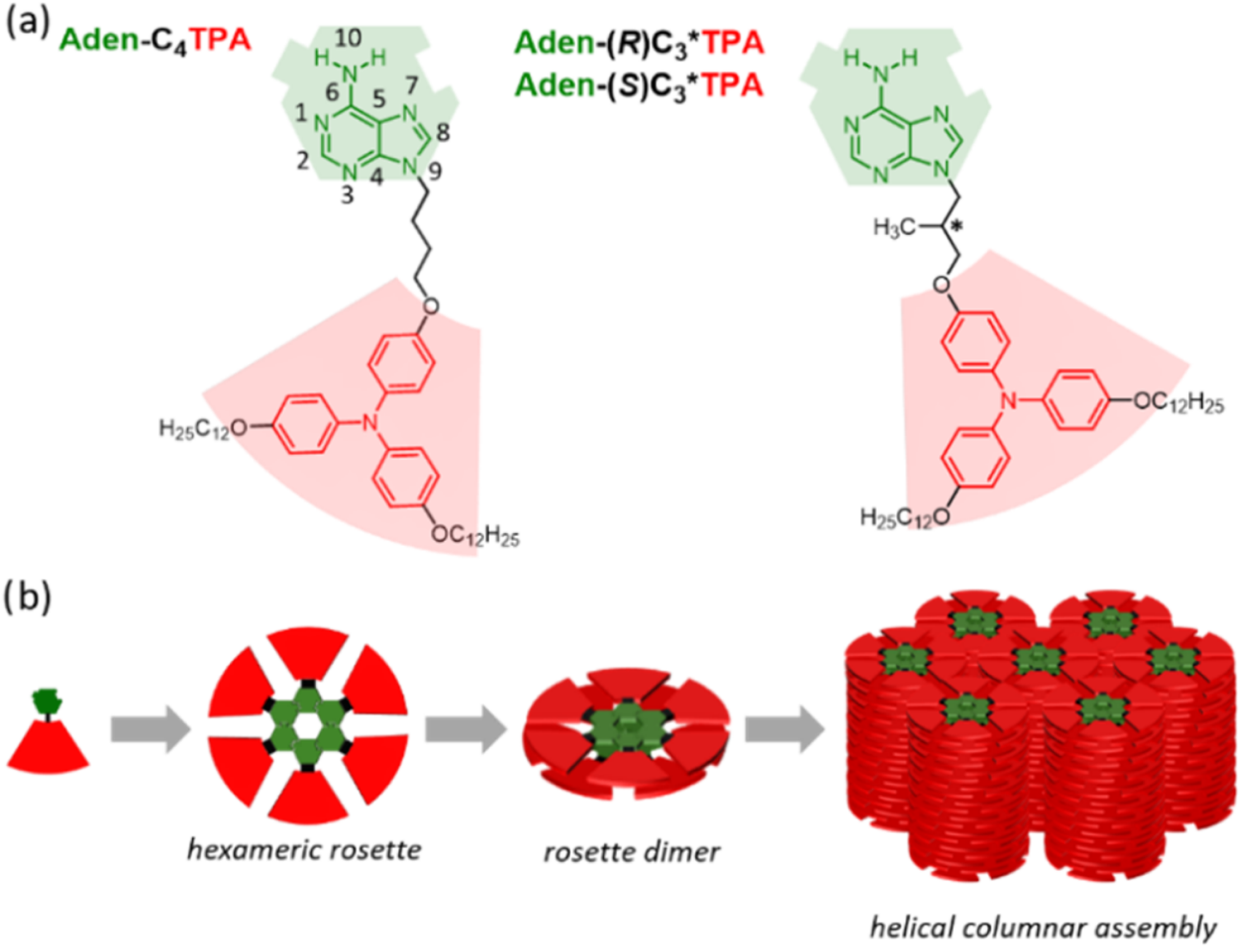
(a) Chemical structures of adenine TPA-containing derivatives. (b) Schematic illustration of their hierarchical self-assembly.

## Results and Discussion

### Synthesis and Characterization

Details of the synthesis and analytical data are given in the Experimental Section and in the Supporting Information (Scheme S1 and Figures S1–S3). **Aden-C**_**4**_ **TPA**, **Aden-**(***R***)**C**_**3**_ *TPA, and **Aden-**(***S***)**C**_**3**_ *TPA were synthesized through a *N*-alkylation reaction between adenine and TPA derivatives modified with bromide (**4**) or tosylate (**6**) aliphatic spacers in the presence of NaH.^30^ Selective *N*-alkylation at the N9 nitrogen of adenine was confirmed by two-dimensional heteronuclear multiple bond correlation (HMBC) NMR experiments (Figure S4). Fourier Transform Infrared (FTIR) spectra of the final compounds showed N–H vibrations related to the formation of hydrogen bonds (see below).

The required TPA units were prepared starting from a benzyl-protected phenolic TPA derivative with two dodecyloxy substituents (**2**), which was synthesized through a Buchwald-Hartwig coupling between 1-dodecyloxy-4-iodobenzene (**1**), prepared from *p*-iodophenol, and 4-benzyloxyaniline.^29^ The benzyl-protecting group was removed via catalytic hydrogenation in the presence of Pd/C to give phenol derivative **3**. The *n*-C_4_H_8_ linear spacer was introduced by a reaction of **3** with 1,4-dibromobutane to give bromide intermediate **4**. The chiral spacers were introduced by reaction of **3** with both enantiomers of 3-bromo-2-methyl-1-propanol yielding **5­(** ***R*** **)** and **5­(** ***S*** **)** and subsequent tosylation of the hydroxyl group to provide the chiral intermediates **6­(** ***S*** **)** and **6­(** ***R*** **)**, respectively.

### Phase Behavior

The as-obtained compounds were crystalline solids, as confirmed by the first heating cycle of differential scanning calorimetry (DSC) studies. However, a peculiar LC behavior emerged for all the compounds on cooling from the isotropic liquid state, and it was maintained in subsequent cooling–heating cycles (Table 1 and Figures S5–S7).

**Table 1:** Phase Behavior Determined by DSC and Lattice Parameter Measured by XRD

compound	thermal properties^a^ *T* (^o^C), [Δ*H* (kJ/mol)]	hexagonal lattice parameter *a* (Å)
**Aden-C_4_TPA** ^b^	Col_h_^hel^ 27 [0.7] Col_h_ 63 [1.0] I	52.1 (r.t.)
		47.4 (45 °C)
Aden(*R*)-C_3_*TPA ^c^	Col_h_^hel^ 59 [9.5] I	51.0 (r.t.)
Aden-(*S*)C_3_*TPA ^c^	Col_h_^hel^ 59 [9.2] I	51.0 (r.t.)

a Temperatures were taken at the maximum of the peak, and enthalpies were measured from optimum heating cycle conditions.

b Second heating process at 1 °C/min.

c Third heating process recorded at 5 °C/min after 24 h at r.t. Col_h_^hel^: helical hexagonal columnar mesophase, Col_h_: hexagonal columnar mesophase, I: isotropic liquid.

Polarized optical microscopy (POM) observations revealed that, on cooling from the isotropic liquid, compound **Aden-C**_**4**_ **TPA** exhibited a birefringent polygonal fan-shaped texture (45 °C in Figure 2a) typical of a hexagonal columnar mesophase (Col_h_ phase). Upon slow cooling to room temperature (rt), this texture evolved to a slightly different polygonal texture with transversal stripes (room temperature in Figure 2a), which indicates a change in the columnar organization (Col_h_^hel^ phase). This is consistent with the transitions observed in the DSC thermogram (Figure S5b) and the X-ray diffraction (XRD) results to be discussed later. For compounds with chiral spacers, **Aden-**(***R***)**C**_**3**_ *TPA and **Aden-**(***S***)**C**_**3**_ *TPA (Figure 2b), only one mesophase (Col_h_^hel^ phase) was observed on cooling at around 45 °C (Figures S6 and S7). The kinetics of formation of the Col_h_^hel^ phase was slow, and it was fully formed after standing at rt for 24 h. Then, on heating, the mesophase changed to isotropic liquid at 59 °C.

**Figure 2:**
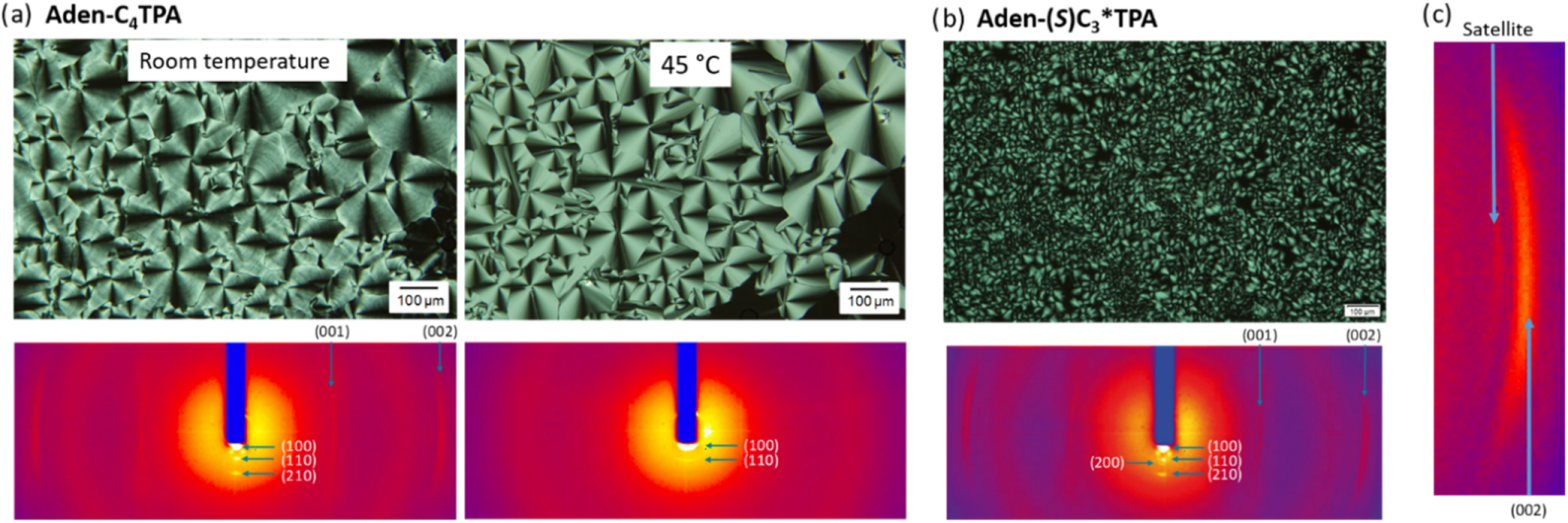
(a) Textures observed by POM and XRD diffraction patterns registered for **Aden-C_4_TPA** at r.t. and 45 °C; (b) texture observed by POM and XRD diffraction pattern registered on cooling for Aden-(*S*)­C_3_*TPA at r.t. (c) Outermost maxima of the XRD pattern of Aden-(*S*)­C_3_*TPA indicating the (002) maximum and the satellite.

### XRD in Oriented Samples

Phases denoted as Col_h_^hel^ and Col_h_ were determined from XRD experiments carried out for oriented samples (Figure 2). In the low-angle region, the r.t. XRD patterns of **Aden-C**_**4**_ **TPA** and **Aden-(** ***S*** **)­C**_3_^*^ **TPA** show three diffraction arcs reinforced perpendicular to the alignment direction related to distances with a ratio 1:1/√3:1/√7 for **Aden-C**_**4**_ **TPA** and 1:1/√3:1/2:1/√7 for **Aden-(** ***S*** **)­C**_3_^*^ **TPA**. These reflections were assigned to the indexes (100), (110), (210) and (100), (110), (200), and (210), respectively, of a hexagonal lattice. In the high-angle region, a diffuse broad reflection is observed at 4.5 Å due to the molten alkyl chains, confirming the liquid crystalline behavior. For both materials, two reflections are observed along the alignment direction corresponding to indexes (001) and (002) and distances 7.7 and 3.8 Å, respectively. A calculation of 6 molecules per column section was made from the measured density values (0.98 g/cm^3^ for **Aden-C**_**4**_ **TPA** and 0.93 g/cm^3^ **Aden-(** ***S*** **)­C**_3_^*^ **TPA**), the lattice parameter *a* (Table 1) and the strongest reflection *d*(002) = 3.8 Å, which is a typical stacking distance for columnar LC phases, and half the *d*(001) = 7.7 Å distance (see Supporting Information, XRD studies section). Both reflections suggest the formation of a rosette-like hexameric complex built through H-bonding interactions between adenines (see below), which in turn form column strata of two stacked rosettes (Figure 1b). The relative arrangement of the rosettes within the dimer is proposed as a 30° rotation, as supported by the XRD simulations discussed below. This 30° rotation represents the maximum contrast within the cell and, therefore, the maximum intensity for the (001) reflection, which would disappear if the rotation angle were zero.

Furthermore, two satellites of the main reflection (002) are observed at both sides of the main reflection (Figure 2c), and this is consistent with modulation λ of the structure along the columnar axis. Considering the distance (in reciprocal space) between reflection (002) and the satellite, the wavelength of the modulation was estimated to be λ = 81.9 Å for **Aden-C**_**4**_ **TPA** and 76.3 Å for **Aden-(** ***S*** **)­C**_3_^*^ **TPA**, which corresponds to approximately 10.6 and 9.9 times, respectively, the stacking distance of 7.7 Å. Taking into account the *C*_6_ symmetry of the rosette-shaped hexameric complex and assuming that the modulation consists of a rotation of the dimer around the (001) direction, it will have rotated 60° along the distance λ. This results in a rotation angle between one dimer and the adjacent dimer of α = 5.6° for **Aden-C**_**4**_ **TPA** and 6.0° for **Aden-(** ***S*** **)­C**_**3**_ *TPA. A set of four symmetry-related small-angle reflections not contained in the (hk0) equatorial plane are observed in both materials at rt (Figure 2a,b). They indicate an interlayer distance of about 35 Å along a direction that makes an angle of about 15° respect to the equator and represent some characteristic distance between the interrelated helically distorted columns.

The high-temperature mesophase of **Aden-C**_**4**_ **TPA** was studied by XRD at 45 °C. A Col_h_ phase was determined from the two reflections in the small-angle region related to distances with a ratio of 1:1/√3. These reflections were assigned to the indexes (100) and (110) of a hexagonal lattice (Figure 2a) with a lattice parameter of 47.4 Å, which is smaller than that at room temperature (52.1 Å). Besides, the reflections related with the helical arrangement disappeared as well as the reflections at 7.7 and 3.8 Å related with a rosette dimer forming the column strata. These results are consistent with an irregular stacking of the hexads so that there is enough space between the disks for the peripheral chains to fold inward into the column, thus explaining the shortening of the lattice parameter.

### Discussion of the Self-Assembly Model Based on a Combination of Theoretical Calculations and XRD and FTIR Studies

Taking as inspiration theoretical studies previously reported for adenine tetrads, there are three different possibilities for the self-assembly of adenine depending on which aromatic nitrogen stablishes H-bonding interaction with the −NH_2_ group, either N1, N3, or N7.^23,24^ Accordingly, three possible hexamers can be envisaged, A_6_-N1, A_6_-N3, and A_6_-N7, as depicted in Figure 3a.

**Figure 3:**
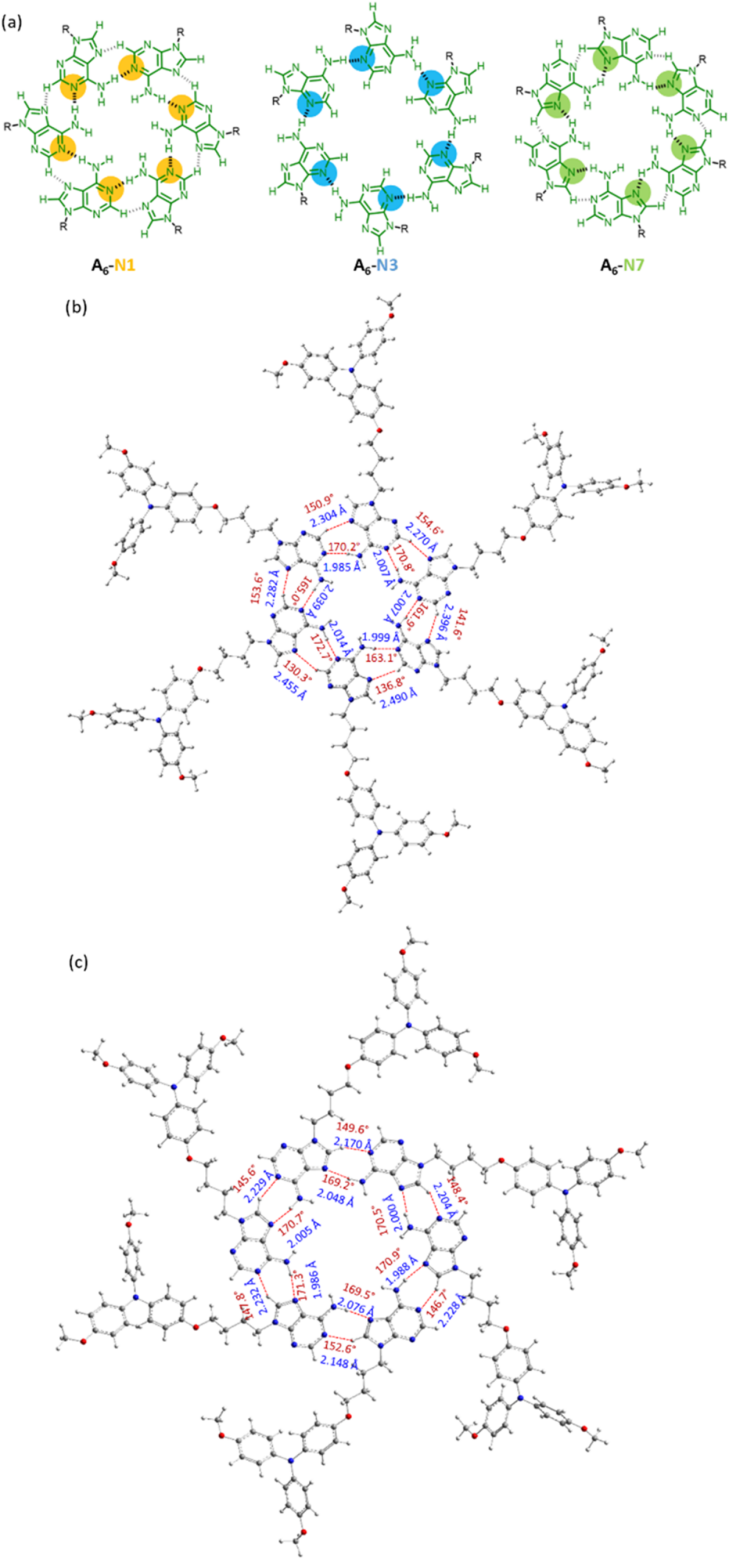
(a) Possible adenine hexameric complexes defined according to reported adenine tetrads.^23,24^ Optimized structure of (b) A_6_-N1 (R = C_4_TPAOCH_3_) complex, and (c) A_6_-N7 (R = C_4_TPAOCH_3_) complex, including H-bond distances and angles calculated at the ωB97xD/6–31G* level of theory. Note that the long peripheral tails of **Aden-C_4_TPA** were replaced with methoxy groups.

In order to shed light onto the possible structure, we first undertook quantum chemical calculations of the three adenine hexamers (R = H) at the ωB97xD/6–31G* level of theory (Figures S8 and S9), which led to discard A_6_-N3. Subsequently, theoretical calculations were performed for a model complex of **Aden-C**_**4**_ **TPA**, in which the long peripheral tails were replaced by methoxy groups in order to reduce the number of atoms and make the system computationally affordable. The results supported the formation of both A_6_-N1 (Figure 3b) and A_6_-N7 (Figure 3c) hexamers (R = C_4_TPAOCH_3_).

The optimized geometries confirmed the existence of weak hydrogen bonds between the hydrogen attached to aromatic carbon C2 and nitrogen N7 in A_6_-N1, and also between the hydrogen attached to aromatic carbon C8 and nitrogen N1 in the A_6_-N7 hexamer. Nevertheless, the A_6_-N7 hexamer was found to be 16.5 kcal/mol energetically more favorable than the A_6_-N1 hexamer (Table S1), in concordance with the higher directionality of the hydrogen bonds showing shorter distances and angles closer to 180° for the A_6_-N7 hexamer than for A_6_-N1 (Figure 3b,c). Indeed, for the A_6_-N7 hexamer, the average value of the angles calculated for internal hydrogen bonds is 170.4 and 148.5° for external bonds, with average bond distances of 2.017 and 2.212 Å, respectively. The average value of the angles calculated for A_6_-N1 hexamer is 167.3° for internal hydrogen bonds and 143.6° for external hydrogen bonds, while the average distances calculated for these bonds are 2.009 and 2.366 Å, respectively.

According to these results, the optimized geometry for hexamer A_6_-N7 (R = C_4_TPAOCH_3_) was selected to obtain simulated XRD patterns (Figures 4 and S10) using the algorithm developed by Alenaizan et al.^31^ Figure 4 presents the good fit between the experimental XRD pattern of **Aden-C**_**4**_ **TPA** at r.t. and the simulated XRD pattern of an idealized helical column, which was built from dimers rotated by −5.6° along the column axis. Each dimer is formed by two optimized A_6_-N7 (R = C_4_TPAOCH_3_) hexamers, which are mutually rotated 30° to avoid steric hindrance between peripheral units. Similar results were obtained for **Aden-(** ***R*** **)­C**_**3**_ *TPA (Figure S11).

**Figure 4:**
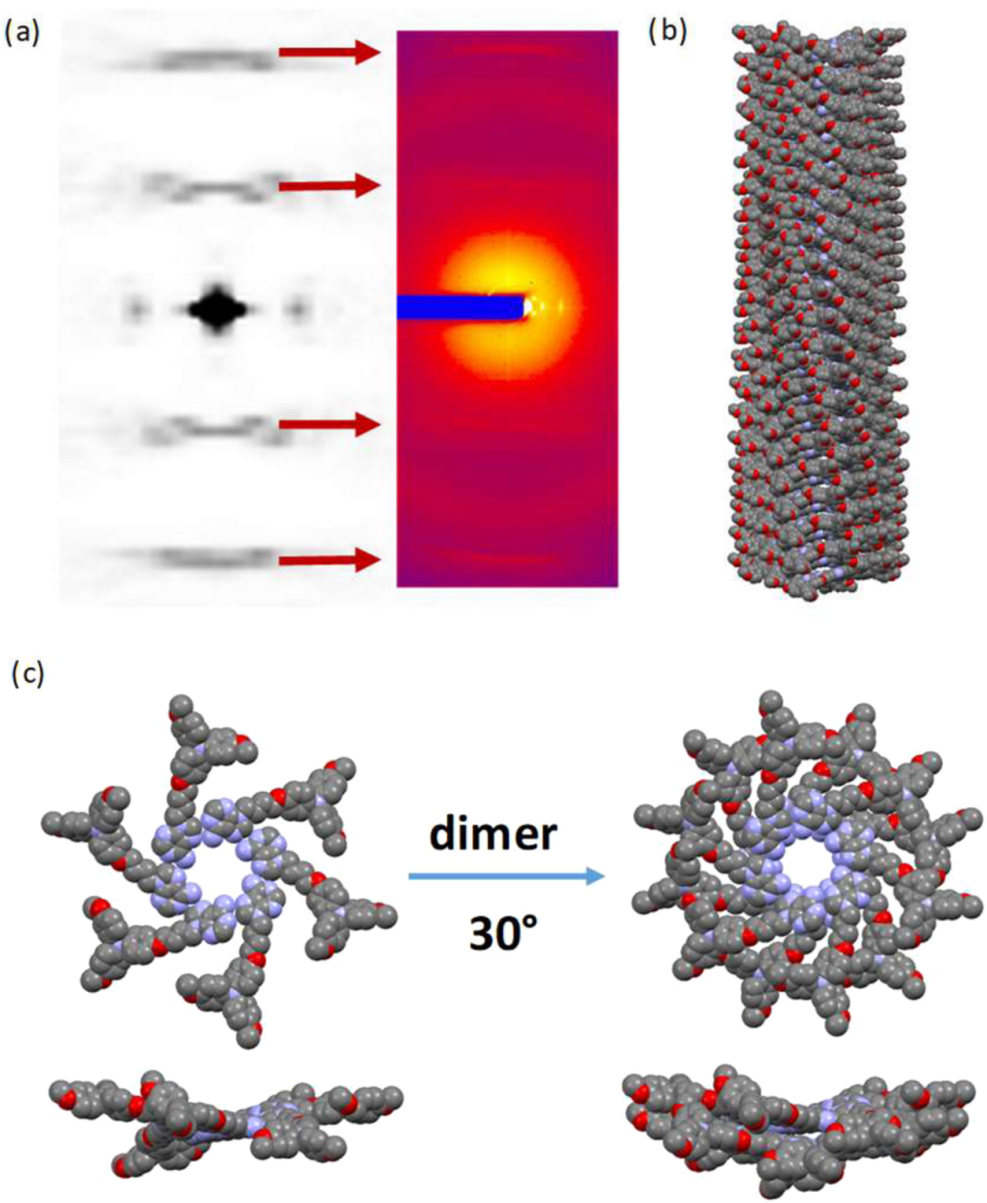
(a) Simulated XRD pattern for an idealized helical column of A_6_-N7 (R = C_4_TPAOCH_3_) consisting of 20 dimers rotated −5.6° (left), and its comparison with the experimental XRD pattern of **Aden-C_4_TPA** at r.t. (right). (b) Helical column obtained from the simulation. (c) Each dimer is made of two stacked hexameric rosettes mutually rotated by 30°.

The directionality of the intermolecular hydrogen bonds formed between adenine units was studied experimentally by FTIR spectroscopy in mechanically aligned samples at r.t. Successful alignment was achieved, as confirmed by POM (Figure S12). All compounds exhibit significant changes at the NH_2_ stretching (st) and NH_2_ bending (ν) bands involved in hydrogen bonding formation with respect to the angle between the polarizer and the alignment direction (Figures 5a and S13). Particularly interesting is the variation of the asymmetric (ν_asym_) and symmetric (ν_sym_) NH_2_ bands. The intensity of the band related to ν_asym_ (≈3300 cm^–1^) is maximum at 0° and decreases as the angle increases, indicating that this vibration occurs with a significant component in the direction of the column axes, perpendicular to the plane of the rosettes. In contrast, the intensity of the band related to ν_sym_ (≈3150 cm^–1^) increases as the angle increases, indicating that this vibration occurs in the plane of the rosettes, perpendicular to the direction of the columns.

**Figure 5:**
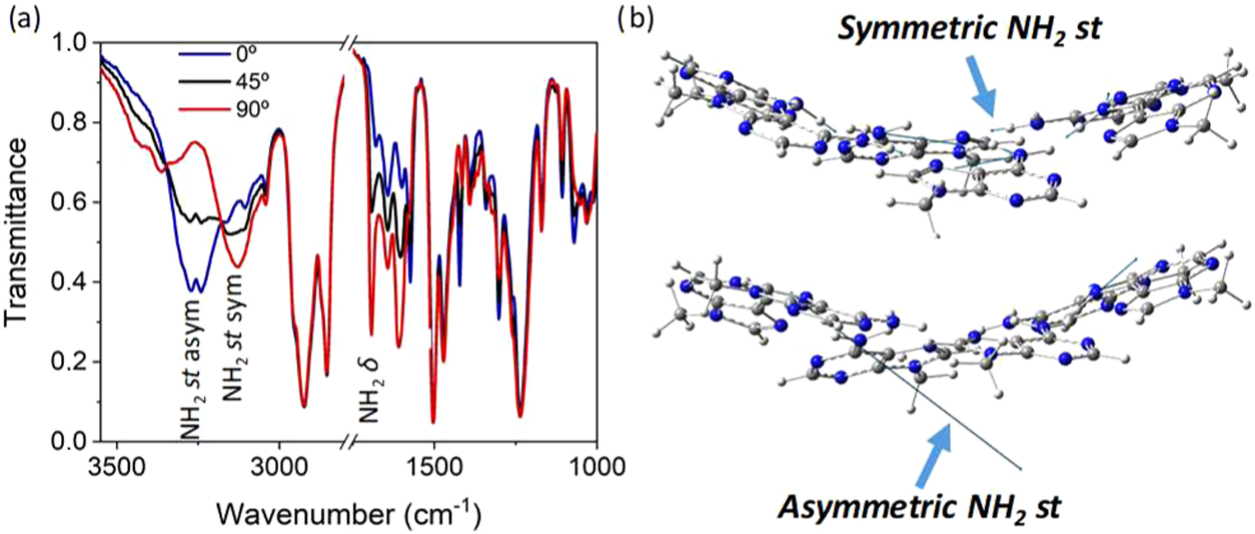
(a) Polarized FTIR spectra (the 0° of the polarizer were made to coincide with the column axes of oriented columns) for **Aden-C_4_TPA**. (b) Snapshot of the videos (SV1 and SV2) recorded for the NH_2_ st vibration modes indicating atomic displacements (blue lines) to represent the direction of the symmetric and asymmetric vibrations in A_6_-N7 calculated at the ωB97xD/6–31G* level of theory.

These observations correlate with the vibrational modes theoretically predicted for the A_6_-N7 (R = H) hexamer (Figures 5b, S14, and Table S2), which showed a different contribution of the hydrogen atoms of the NH_2_ group to ν_sym_ and ν_asym_. Indeed, ν_sym_ shows the greatest contribution from the hydrogen involved in the formation of the hydrogen bond, which shows a vibration direction in the plane of the rosette (Figure S14a and Video SV1). In contrast, the ν_asym_ shows the greatest contribution from the nonbonded hydrogen, with a displacement direction lying out of the plane that contains the adenine hexameric complex (Figure S14b and Video VS2).

These results support the formation for the first time of adenine homo-assemblies with a rosette-like hexameric structure and columnar LC behavior. Unlike previously described homorosettes with columnar LC behavior, such as those based on triazine^32–34^ or barbituric acid,^35–37^ adenine rosettes shown here display different degrees of hierarchical organization, namely dimers and helical stacks, which self-organized into hexagonal columnar LC phases.

### Supramolecular Chirality

The proposed helical model suggests that these nanostructures could serve as an effective platform for inducing supramolecular chirality by transferring molecular chirality to the columnar architecture. The columnar mesophase of the derivatives with a chiral spacer, **Aden-(** ***R*** **)­C**_**3**_ *TPA and **Aden-(** ***S*** **)­C**_**3**_ *TPA, were studied by electronic circular dichroism (CD) and exhibited opposite optical activity (Figures 6 and S15). Three positive bands can be identified that correspond to absorption bands of both the N9-substituted adenine at 260 and 275 nm and TPA at 300 nm (Figures S15a and S16), and this is consistent with both chromophores within a chiral environment. Furthermore, the band at 360 nm is very characteristic of TPA units arranged in a helical manner, likely driven by a frozen chiral-propeller conformation.^38–40^ Variable temperature CD spectra show that the optical activity of the material only appears in the mesophase (Figure 6) and vanishes in the isotropic liquid, assessing that CD response is due to supramolecular chirality rather than to molecular chirality. This supports the helical model of the column, which is likely originated from a helical disposition of rosette dimers, as observed by XRD, biased toward a chiral sense due to the chirality of the flexible spacer between adenine and TPA.

**Figure 6:**
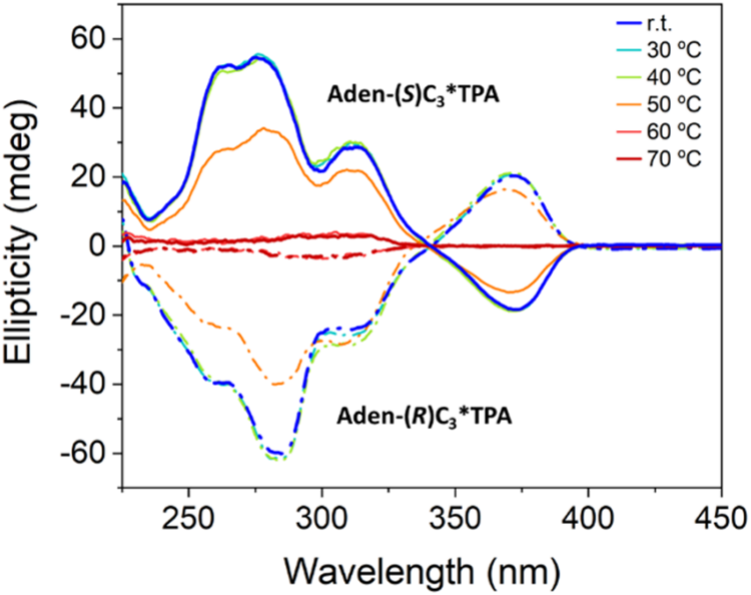
CD spectra recorded for Aden-(*S*)­C_3_*TPA (solid lines) and Aden-(*R*)­C_3_*TPA (dashed dotted lines) at variable temperature.

### Hole Transport

The presence of peripheral electron-donor TPA groups endowed these supramolecular systems with high highest occupied molecular orbital (HOMO) levels (Figure S17 and Table S3), which are effectively localized in the TPA units, as determined by density functional theory (DFT) calculations (Figure S18 and Table S4). This prompted the study of the semiconducting behavior of these new materials. The hole transport properties were measured via the space charge-limited current (SCLC) method at r.t. by preparing devices for hole mobility using solution-processed thin films (Figure S19).^41^ These films were thermally treated by slow cooling at r.t. to enhance column alignment perpendicular to the substrate.^28^ All the compounds showed similar hole mobility values with two distributions; the highest values ranged around 10^–3^ cm^2^·s^–1^·V^–1^, while the lowest values were around 10^–5^ cm^2^·s^–1^·V^–1^ (Figures S20 and S21). These values are on the order of those measured for previous TPA-containing columnar LCs. The distribution of mobility value within two regimes represents the strong correlation between charge mobility and column orientation with respect to electrode surfaces. The higher mobility values are related with areas of the sample with a more uniform column orientation closer to the normal to the electrodes. In such areas, percolation pathways for charge transport along column axes can be obtained and the measured mobility reflects the faster charge motion due to intracolumnar π–π overlap. In other areas of samples, column orientation is less uniform or not along the normal to the electrode surfaces, i.e., the direction along which mobility is measured. In this case, charge transport implies intercolumnar charge transfer, a much slower process, and mobility decreases by orders of magnitude.

## Conclusions

We here report on a novel design of mesogenic molecules centered on adenine, specifically N9 adenines substituted with TPA units connected via an aliphatic spacer. As unconventional mesogens, their organization in columnar mesophases has been studied in depth by experimental techniques (POM, DSC, XRD, and polarized FTIR) with the strong support of theoretical calculations. These molecules form unprecedented supramolecular entities consisting of adenine hexameric rosettes formed by hydrogen bonding. At a higher level of hierarchy, these rosettes form dimers that organize into chiral columns, even with nonchiral molecules. These columns are further arranged into a highly ordered hexagonal columnar mesophase, which remains stable at r.t. Introducing chiral molecules reveals that both adenine and TPA exist within a chiral environment. Furthermore, the resulting columnar organizations exhibit a promising hole transport behavior. This distinctive molecular arrangement organization paves the way for the creation of complex, functional systems with low synthetic effort thanks to the hierarchical assembly of simple adenine-based molecules, offering exciting potential for future materials science and nanotechnology applications.

## Experimental Section

### Experimental Techniques

^1^H NMR and ^13^C NMR spectra were acquired on a Bruker AV400 spectrometer. The experiments were performed at room temperature in different deuterated solvents (CDCl_3_ and acetone-d_6_). Chemical shifts are given in ppm relative to tetramethylsilane (TMS), and the solvent residual peak was used as an internal standard.

FTIR spectra were recorded on a Bruker Vertex 70 FTIR spectrometer. Samples were prepared on KBr pellets with a concentration of the product of 1–2% (w/w). For polarized FTIR experiments, a ZnSe polarizer was used, and the materials were placed between two KBr polished IR crystal windows (13 mm diameter × 2 mm thickness).

Mass spectrometry was performed on a Bruker Q-TOF-MS instrument by electrospray ionization (ESI) in positive mode.

Thermal behavior was studied by polarizing optical microscopy using a polarizing optical microscope, an Olympus BX51 equipped with an Olympus DP22 digital camera and a Linkam THMS600 hot stage. Transition temperatures and enthalpies were obtained by differential scanning calorimetry with DSC TA Instruments Q20 and Q2000 calorimeters at heating and cooling rates of 10, 5, or 1 °C min^–1^ and previously calibrated with indium (156.6 °C, 28.71 J g^–1^).

XRD diagrams were recorded using a Stoe Stadivari goniometer equipped with a Genix3D microfocus generator (Xenocs) and a Dectris Pilatus 100 K detector. Monochromatic Cu Kα radiation (λ = 1.5418 Å) was used. Temperature control was achieved using a nitrogen-gas Cryostream controller (Oxford Cryosystems). The samples were held in Lindemann glass capillaries (0.9 mm diameter).

The density of the materials was calculated experimentally by the buoyancy method by suspending the materials in 1 mL of distilled water (ρ = 1.00 g/mL) and adding portions of 20 μL of methanol (ρ = 0.792 g/mL) progressively until the material started floating. Finally, the density of the mixture was calculated with the mass and volume of the mixture.

CD spectra were recorded on a Jasco J-810 spectropolarimeter. For thin film sample preparation, a 100 μL CHCl_3_ solution of the material was spin-coated onto a quartz plate (30 mg/mL at 1500 rpm for 1 min), heated to the isotropic liquid, and slowly cooled to room temperature to promote proper mesophase formation. CD spectra were recorded at different rotation angles around the light beam showed the same trace and were averaged in order to compensate for linear dichroism artifacts (Figure S15b,c). Experiments at variable temperatures were performed by heating the films with a Mettler FP80 HT hot stage.

Cyclic voltammetry experiments were performed using an Autolab PGSTAT204 potentiostat with 0.1 M tetrabutylammonium hexafluorophosphate solutions in dry, oxygen free dichloromethane. Graphite was used as the working electrode, a platinum gauze as the auxiliary electrode, and Ag/AgCl as the reference electrode. Product concentration was 10^–4^ M.

### Computational Details

Full geometry optimization of the ground state was performed using the Gaussian16 (revision A.03) suite of programs.^42^ The ωB97x - D functional^43^ was chosen along with the 6–31G* basis sets. The vibrational modes were calculated to check the absence of imaginary frequencies in A_6_-N1, A_6_-N3, and A_6_-N7.

### General Procedure for the Synthesis of the N9 Substituted Adenines Shown in This Work and Their Characterization Data

NaH (0.03 g, 1.15 mmol) was added to a suspension of adenine (0.13 g, 1 mmol) in dry dimethylformamide under an argon atmosphere. The mixture was stirred at room temperature for 1 h until no more H2 bubbling was observed. After this time, the corresponding bromide (4) or tosylate (6­(*R*) or 6­(*S*)) (1.3 mmol) was added, and the reaction mixture was stirred overnight at room temperature. Then, the solvent was evaporated under reduced pressure, and the resulting crude was suspended in 50 mL of dichloromethane and filtered. The obtained solution was evaporated under reduced pressure, and the residue was purified by flash chromatography starting with dichloromethane as an initial solvent and gradually increasing the polarity to dichloromethane/methanol 98:2.

#### Aden-C_4_TPA

The product was further recrystallized from ethanol, obtaining a white powder. Yield: 46%. ^**1**^ **H NMR** (400 MHz, CDCl_3_, 25 °C, TMS, ppm): δ = 8.36 (s, 1H, ArH), 7.84 (s, 1H, ArH), 6.97–6.90 (m, 6H, ArH), 6.79–6.71 (m, 6H, ArH), 5.54 (s, 2H, NH_2_), 4.30 (t, *J* = 7.2 Hz, 2H, NCH_2_), 3.96 (t, *J* = 6.0 Hz, 2H, OCH_2_), 3.90 (t, *J* = 6.6 Hz, 4H, OCH_2_), 2.16–2.06 (m, 2H, CH_2_), 1.85–1.70 (m, 6H, CH_2_), 1.48–1.17 (m, 36 H, CH_2_), 0.88 (t, *J* = 6.8 Hz, 6H, CH_3_). ^**13**^ **C NMR** (100 MHz, CDCl_3_, 25 °C, TMS, ppm): δ = 155.5 (C*C*NH_2_), 154.7 (O*C*C), 154 (O*C*C), 153.1 (N*C*H), 150.4 (N*C*C), 142.5 (N*C*C), 141.9 (N*C*C), 140.7 (N*C*H), 125.1 (C*C*H), 124.7 (C*C*H), 119.9 (N*C*C), 115.3 (C*C*H), 115.2 (C*C*H), 68.5 (OCH_2_), 67.6 (OCH_2_), 43.8 (NCH_2_), 32.1 (CH_2_), 29.8 (CH_2_), 29.8 (CH_2_), 29.8 (CH_2_), 29.7 (CH_2_), 29.6 (CH_2_), 29.5 (CH_2_), 29.5 (CH_2_), 27.3 (CH_2_), 26.6 (CH_2_), 26.2 (CH_2_), 22.8 (CH_2_), 14.3 (CH_3_). **FTIR** (KBr, cm^–1^): 3287 (br) (N–H *st*), 3124 (br) (N–H *st*), 2923 (vs) (Csp^3^-H), 2852 (vs) (Csp^3^-H), 1680 (s) (N–H δ), 1602 (s) (C–N *st*), 1572 (w) (C–C_Ar_), 1504 (vs) (C–C_Ar_), 1237 (vs) (C–O *st*). **HRMS** (ESI+) *m*/*z* calcd for C_51_H_74_N_6_O_3_^+^: 818.5822 [*M*]^+^; found: 818.5812 [*M*]^+^.

Aden-(*R*)­C_3_*TPA and Aden-(*S*)­C_3_*TPA. The products were recrystallized from methanol, obtaining sticky colorless solids. Compound **Aden-(** ***R*** **)­C**_**3**_ *TPA yield: 39%. Compound **Aden-(** ***S*** **)­C**_**3**_ *TPA yield: 41%. ^**1**^ **H RMN** (400 MHz, CDCl_3_, 25 °C, TMS, ppm): δ = 8.37 (s, 1H, ArH), 7.76 (s, 1H, ArH), 6.98–6.89 (m, 6H, ArH), 6.81–6.70 (m, 6H, ArH), 5.56 (s, 2H, NH_2_), 4.37 (dd, *J* = 14, 7.2 Hz, 1H, NCH_2_), 4.26 (dd, *J* = 14, 7.2 Hz, 1H, NCH_2_), 3.91 (t, *J* = 6.6 Hz, 4H, OCH_2_), 3.82–3.72 (m, 2H, OCH_2_), 2.68–2.55 (m, 1H, CH), 1.81–1.70 (m, 4H, CH_2_), 1.50–1.20 (m, 36H, CH_2_), 1.10 (d, *J* = 6.9 Hz, 3H, CH_3_), 0.88 (t, *J* = 6.9 Hz, 6H, CH_3_). ^**13**^ **C RMN** (100 MHz, CDCl_3_, 25 °C, TMS, ppm): δ = 155.5 (C*C*NH_2_), 154.7 (O*C*C), 153.7 (O*C*C), 153.2 (N*C*H), 150.6 (N*C*C), 142.7 (N*C*C), 141.8 (N*C*C), 141.4 (N*C*H), 125.1 (CCH), 124.5 (CCH), 119.7 (NCC), 115.3 (CCH), 115.1 (CCH), 69.7 (OCH_2_), 68.4 (OCH_2_), 46.6 (NCH_2_), 34.2 (CH), 32 (CH_2_), 29.8 (CH_2_), 29.7 (CH_2_), 29.6 (CH_2_), 29.5 (CH_2_), 29.4 (CH_2_), 26.2 (CH_2_), 22.8 (CH_2_), 15 (CH_3_), 14.3 (CH_3_). **FTIR** (KBr, cm^–1^): 3307 (br) (N–H *st*), 3149 (br) (N–H *st*), 2933 (vs) (Csp^3^-H), 2855 (vs) (Csp^3^-H), 1644 (s) (N–H δ), 1601 (s) (C–N *st*), 1575 (w) (C–C_Ar_), 1504 (vs) (C–C_Ar_), 1236 (vs) (C–O *st*). **HRMS** (ESI+) *m*/*z* calcd for C_51_H_75_N_6_O_3_^+^: 819.5901 [*M* + H]^+^; found: (*R*): 819.5881 [*M* + H]^+^, (S): 819.5871 [*M* + H]^+^.

## Supplementary Material







## Notes

Synthetic route and full experimental procedures and characterization data; NMR spectra; DSC thermograms; additional information about: XRD studies, adenine hexamer optimization, model for the Col_h_^hel^ phase, H-bond studies by FTIR, CD spectra, electrochemical properties, energy levels, and charge mobility measurements (PDF)
Atomic displacement for the symmetric NH_2_ st vibrational mode (SV2 video) (MP4)
Atomic displacement for the asymmetric NH_2_ st vibrational mode (SV2 video) (MP4)

## Abbreviations

LC: liquid crystal
Aden: adenine
TPA: triphenylamine
NMR: nuclear magnetic resonance
FTIR: Fourier transform infrared
HRMS: high-resolution mass spectrometry
POM: polarized optical microscopy
DSC: differential scanning calorimetry
XRD: X-ray diffraction
CD: circular dichroism
r.t.: room temperature

## References

[1] Yashima E., Ousaka N., Taura D., Shimomura K., Ikai T., Maeda K. (2016). Supramolecular Helical Systems: Helical
Assemblies of Small Molecules, Foldamers, and Polymers with Chiral
Amplification and Their Functions. Chem. Rev..

[2] Shimizu T., Ding W., Kameta N. (2020). Soft-Matter
Nanotubes: A Platform
for Diverse Functions and Applications. Chem.
Rev..

[3] Uchida J., Soberats B., Gupta M., Kato T. (2022). Advanced Functional
Liquid Crystals. Adv. Mater..

[4] Bisoyi H. K., Li Q. (2022). Liquid Crystals: Versatile Self-Organized Smart Soft Materials. Chem. Rev..

[5] Hashim P. K., Bergueiro J., Meijer E. W., Aida T. (2020). Supramolecular Polymerization:
A Conceptual Expansion for Innovative Materials. Prog. Polym. Sci..

[6] Ariga K., Shrestha L. K. (2019). Supramolecular nanoarchitectonics
for functional materials. APL Mater..

[7] Lugger S. J. D., Houben S. J. A., Foelen Y., Debije M. G., Schenning A. P. H. J., Mulder D. J. (2022). Hydrogen-Bonded Supramo-lecular Liquid Crystal Polymers:
Smart Materials with Stimuli-Responsive, Self-Healing, and Recyclable
Properties. Chem. Rev..

[8] Giménez, R. ; Martínez-Bueno, A. ; Sierra, T. Noncovalent Design of Columnar LCs on the Way to Nanostructured Functional Materials. In Supramolecular Nanotechnology; Azzaroni, O. ; Conda-Sheridan, M. , Eds.; Wiley-VCH:: Weinheim, 2023; Vol. 1, pp 425–446.

[9] Sahoo D., Peterca M., Percec V. (2024). Hierarchical Self-Organization
and
Disorganization of Helical Supramolecular Columns Mediated by H-Bonding
and Shape Complementarity. J. Am. Chem. Soc..

[10] Adhikari B., Lin X., Yamauchi M., Ouchi H., Aratsu K., Yagai S. (2017). Hydrogen-bonded
rosettes comprising π-conjugated systems as building blocks
for functional one-dimensional assemblies. Chem.
Commun..

[11] del
Prado A., González-Rodríguez D., Wu Y.-L. (2020). Functional Systems Derived from Nucleobase Self-assembly. ChemistryOpen.

[12] Krissanaprasit A., Key C. M., Pontula S., LaBean T. H. (2021). Self-Assembling
Nucleic Acid Nanostructures Functionalized with Aptamers. Chem. Rev..

[13] Yao N., Wu J., Liu G., Hua Z. (2024). Bioinspired and biomimetic
nucleobase-containing
polymers: the effect of selective multiple hydrogen bonds. Chem. Sci..

[14] Mayoral M. J., Bilbao N., González-Rodríguez D. (2016). Hydrogen-Bonded
Macrocyclic Supramolecular Systems in Solution and on Surfaces. ChemistryOpen.

[15] Davis J. T. (2004). G-Quartets
40 Years Later: From 5′-GMP to Molecular Biology and Supramolecular
Chemistry. Angew. Chem., Int. Ed..

[16] Sreenivasachary N., Lehn J.-M. (2005). Gelation driven
component selection in the generation
of constitutional dynamic hydrogels based on guanine-quartet formation. Proc. Natl. Acad. Sci. U.S.A..

[17] Wu Y.-L., Brown K. E., Gardner D. M., Dyar S. M., Wasielewski M. R. (2015). Photoinduced
Hole Injection into a Self-Assembled π-Extended G-Quadruplex. J. Am. Chem. Soc..

[18] Spada G. P., Gottarelli G. (2004). The Disclosure of the Stepwise Supramolecular Organization
of Guanosine Derivatives: Serendipity or Programmed Design?. Synlett.

[19] Mariani P., Mazabard C., Garbesi A., Spada G. P. (1989). A study of the structure
of the lyomesophases formed by the dinucleoside phosphate d­(GpG).
An approach by x-ray diffraction and optical microscopy. J. Am. Chem. Soc..

[20] Huang Z., Li X., Chen M., Liu Y., Sun X., Song A., Hao J. (2019). Guanosine-based thermotropic liquid crystals with tunable phase structures
and ion-responsive properties. J. Colloid Interface
Sci..

[21] Gan K. P., Yoshio M., Sugihara Y., Kato T. (2018). Guanine–oligothiophene
conjugates: liquid-crystalline properties, photoconductivities and
ion-responsive emission of their nanoscale assemblies. Chem. Sci..

[22] Escaja N., Mir B., Garavís M., González C. (2022). Non-G Base
Tetrads. Molecules.

[23] Gu J., Leszczynski J. (2001). The structure,
stability, H-bonding pattern, and electrostatic
potential of adenine tetrads. Chem. Phys. Lett..

[24] Szatylowicz H., Marek P. H., Stasyuk O. A., Krygowski T. M., Solà M. (2020). Substituted adenine quartets: interplay
between substituent
effect, hydrogen bonding, and aromaticity. RSC
Adv..

[25] Patel P. K., Koti A. S. R., Hosur R. V. (1999). NMR studies
on truncated sequences
of human telomeric DNA: Observation of a novel A-tetrad. Nucleic Acids Res..

[26] Liu H., Wang R., Yu X., Shen F., Lan W., Haruehanroengra P., Yao Q., Zhang J., Chen Y., Li S. (2018). High-resolution DNA quadruplex structure containing
all the A-, G-, C-, T-tetrads. Nucleic Acids
Res..

[27] Itahara T., Yokogawa Y. (2007). Self-organization of
adenine and thymine derivatives
in thermotropic liquid crystal. J. Mol. Struct..

[28] Martínez-Bueno A., Martín S., Ortega J., Folcia C. L., Termine R., Golemme A., Giménez R., Sierra T. (2024). Effect of Hydrogen
Bonding and Chirality in Star-Shaped Molecules with Peripheral Triphenylamines:
Liquid Crystal Semiconductors and Gels. Chem.
Mater..

[29] Feringán B., Termine R., Golemme A., Granadino-Roldán J. M., Navarro A., Giménez R., Sierra T. (2021). Triphenylamine- and
triazine-containing hydrogen bonded complexes: liquid crystalline
supramolecular semiconductors. J. Mater. Chem.
C.

[30] Spijker H. J., Dirks A. J., van Hest J. C. M. (2005). Unusual
rate enhancement in the thymine
assisted ATRP process of adenine monomers. Polymer.

[31] Alenaizan A., Borca C. H., Karunakaran S. C., Kendall A. K., Stubbs G., Schuster G. B., Sherrill C. D., Hud N. V. (2021). X-ray Fiber Diffraction
and Computational Analyses of Stacked Hexads in Supramolecular Polymers:
Insight into Self-Assembly in Water by Prospective Prebiotic Nucleobases. J. Am. Chem. Soc..

[32] Pisula W., Tomović Ž., Wegner M., Graf R., Pouderoijen M. J., Meijer E. W., Schenning A. P. H. J. (2008). Liquid
crystalline hydrogen bonded oligo­(p-phenylenevinylene)­s. J. Mater. Chem..

[33] Vera F., Barberá J., Romero P., Serrano J. L., Ros M. B., Sierra T. (2010). Orthogonal Action of Noncovalent Interactions for Photoresponsive
Chiral Columnar Assemblies. Angew. Chem., Int.
Ed..

[34] Feringán B., Folcia C. L., Termine R., Golemme A., Granadino-Roldán J. M., Navarro A., Serrano J. L., Giménez R., Sierra T. (2018). Inspecting the Electronic Architecture and Semiconducting
Properties of a Rosette-Like Supramolecular Columnar Liquid Crystal. Chem. - Eur. J..

[35] Yagai S., Kinoshita T., Kikkawa Y., Karatsu T., Kitamura A., Honsho Y., Seki S. (2009). Interconvertible Oligothiophene Nanorods
and Nanotapes with High Charge-Carrier Mobilities. Chem. - Eur. J..

[36] Yagai S., Suzuki M., Lin X., Gushiken M., Noguchi T., Karatsu T., Kitamura A., Saeki A., Seki S., Kikkawa Y., Tani Y., Nakayama K.-i. (2014). Supramolecular Engineering
of Oligothiophene Nanorods without Insulators: Hierarchical Association
of Rosettes and Photovoltaic Properties. Chem.
- Eur. J..

[37] Ouchi H., Lin X., Kizaki T., Prabhu D. D., Silly F., Kajitani T., Fukushima T., Nakayama K.-i., Yagai S. (2016). Hydrogen-bonded oligothiophene
rosettes with a benzodithiophene terminal unit: self-assembly and
application to bulk heterojunction solar cells. Chem. Commun..

[38] Osypenko A., Moulin E., Gavat O., Fuks G., Maaloum M., Koenis M. A. J., Buma W. J., Giuseppone N. (2019). Temperature
Control of Sequential Nucleation–Growth Mechanisms in Hierarchical
Supramolecular Polymers. Chem. - Eur. J..

[39] Kim K. Y., Kim C., Choi Y., Jung S. H., Kim J. H., Jung J. H. (2018). Helicity
Control of Triphenylamine-Based Supramolecular Polymers: Correlation
between Solvent Properties and Helicity in Supramolecular Gels. Chem. - Eur. J..

[40] Adelizzi B., Filot I. A. W., Palmans A. R. A., Meijer E. W. (2017). Unravelling the
Pathway Complexity in Conformationally Flexible N-Centered Triarylamine
Trisamides. Chem. - Eur. J..

[41] Termine R., Golemme A. (2021). Charge Mobility in Discotic Liquid Crystals. Int. J. Mol. Sci..

[42] Frisch, M. J. ; Trucks, G. W. ; Schlegel, H. B. ; Scuseria, G. E. ; Robb, M. A. ; Cheeseman, J. R. ; Scalmani, G. ; Barone, V. ; Petersson, G. A. ; Nakatsuji, H. ; Li, X. ; Caricato, M. ; Marenich, A. V. ; Bloino, J. ; Janesko, B. G. ; Gomperts, R. ; Mennucci, B. ; Hratchian, H. P. ; Ortiz, J. V. ; Izmaylov, A. F. ; Sonnenberg, J. L. ; Williams-Young, D. ; Ding, F. ; Lipparini, F. ; Egidi, F. ; Goings, J. ; Peng, B. ; Petrone, A. ; Henderson, T. ; Ranasinghe, D. ; Zakrzewski, V. G. ; Gao, J. ; Rega, N. ; Zheng, G. ; Liang, W. ; Hada, M. ; Ehara, M. ; Toyota, K. ; Fukuda, R. ; Hasegawa, J. ; Ishida, M. ; Nakajima, T. ; Honda, Y. ; Kitao, O. ; Nakai, H. ; Vreven, T. ; Throssell, K. ; Montgomery, J. A., Jr. ; Peralta, J. E. ; Ogliaro, F. ; Bearpark, M. J. ; Heyd, J. J. ; Brothers, E. N. ; Kudin, K. N. ; Staroverov, V. N. ; Keith, T. A. ; Kobayashi, R. ; Normand, J. ; Raghavachari, K. ; Rendell, A. P. ; Burant, J. C. ; Iyengar, S. S. ; Tomasi, J. ; Cossi, M. ; Millam, J. M. ; Klene, M. ; Adamo, C. ; Cammi, R. ; Ochterski, J. W. ; Martin, R. L. ; Morokuma, K. ; Farkas, O. ; Foresman, J. B. ; Fox, D. J. Gaussian 16, Revision A.03.; Gaussian, Inc.: Wallingford CT, 2016.

[43] Chai J.-D., Head-Gordon M. (2008). Systematic optimization of long-range corrected hybrid
density functionals. J. Chem. Phys..

