# Supplementary material for: Unveiling Adenine H‑bonded Hexads: Hierarchical Self-Assembly for Helical Columnar Functional Materials
Source: JACS Au. 2025 Jun 19;5(7):3115–24. doi: 10.1021/jacsau.5c00256 (PMC12308428; doi:10.1021/jacsau.5c00256)
Supplement: Supplementary file 1 [file au5c00256_si_001.pdf]

Supporting Information for

Unveiling Adenine H-bonded Hexads: Hierarchical Self-assembly for Helical Columnar Functional Materials.

Alejandro Martínez-Bueno,[a,b] Génesis M. Valencia-Vásconez,[a,b] Roberto Termine,[c] Attilio Golemme,[c] Josu Ortega,[d] César L. Folcia,*[,d] José M. Granadino-Roldán,[e] Amparo Navarro,*[,e] Raquel Giménez*[,a,b,‡] and Teresa Sierra*[,a,b,‡]

a Instituto de Nanociencia y Materiales de Aragón (INMA), CSIC-Universidad de Zaragoza, 50009 Zaragoza, Spain.

b Departamento de Química Orgánica, Facultad de Ciencias, Universidad de Zaragoza, 50009 Zaragoza, Spain.

c CNR-NANOTEC SS di Rende, Dipartimento di Fisica, Università della Calabria, 87036 Rende, Italy.

d Department of Physics, Faculty of Science and Technology, UPV/EHU, 48940 Bilbao, Spain.

e Departamento de Química Física y Analítica, Facultad de Ciencias Experimentales, Universidad de Jaén, Campus Las Lagunillas, 23071 Jaén, Spain.

Index

**Synthesis and characterization**	**S2**
**^{1}H NMR and ^{13}C NMR spectra**	**S7**
**Thermal properties. DSC thermograms**	**S11**
**XRD studies**	**S13**
**Adenine hexamer optimization**	**S14**
**Model for the Col_h^{hel} phase**	**S16**
**Hydrogen bond studies by FTIR**	**S18**
**Chirooptical properties. CD spectra**	**S21**
**Electrochemical properties and energy levels**	**S23**
**Charge mobility measurements**	**S24**

Synthesis and characterization

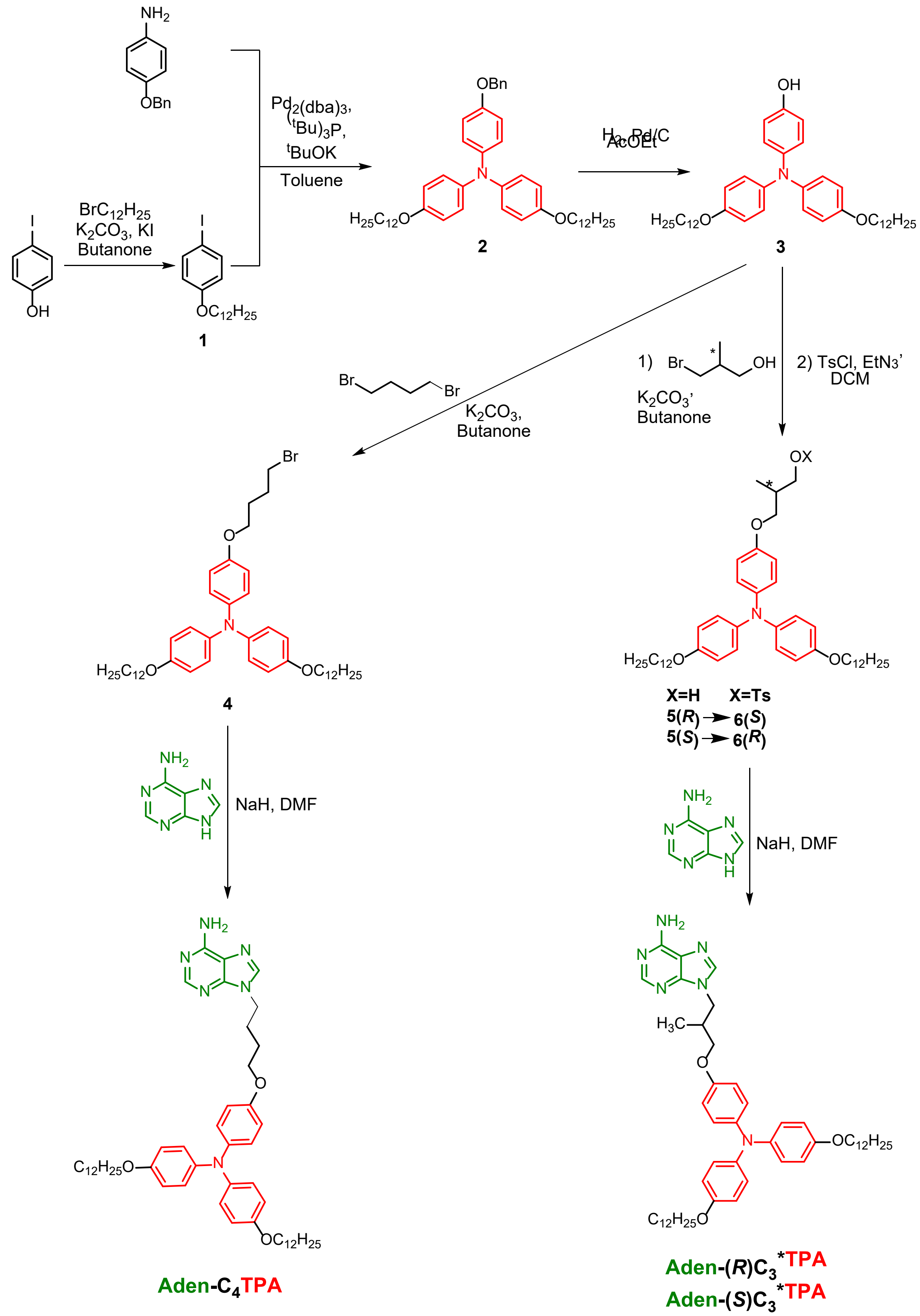

Scheme S1: Synthetic pathways to obtain **Aden-C$_4$TPA**, **Aden-(*R*)C$_3$*TPA** and **Aden-(*S*)C$_3$*TPA**

Compounds **1**, **2** and **3** were synthesized as described previously.[1]

Compound 4

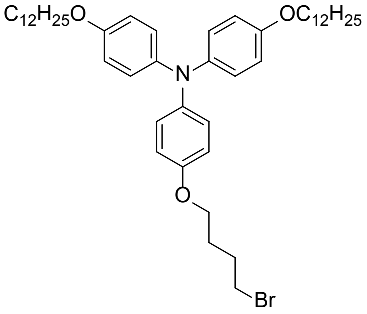

Potassium carbonate (1.4 g, 10 mmol) and 1,4-dibromobuthane (1.7 g, 8 mmol) were added to a solution of compound **3** (1.3 g, 2 mmol) in butanone (80 mL) under an argon atmosphere. The resulted mixture was stirred 16 h under reflux (80 °C). After cooling at room temperature, the mixture was filtered through a celite® pad, the solvent was removed under vacuum and the crude oil was purified by flash chromatography using hexane/ethyl acetate (95:5). The product was obtained as a colorless oil. Yield: 90%.

^{1}H-NMR (400 MHz, [D_6]Acetone, 25°C, TMS, ppm): δ = 6.94 - 6.87 (m, 6H, ArH), 6.86 - 6.80 (m, 6H, ArH), 4.00 (t, *J* = 6.2 Hz, 2H, OCH_2), 3.94 (t, *J* = 6.5 Hz, 4H, OCH_2), 3.59 (t, *J* = 6.7 Hz, 2H, CH_2Br), 2.11 - 2.02 (m, 2H, CH_2), 1.96 - 1.87 (m, 2H, CH_2), 1.80 - 1.70 (m, 4H, CH_2), 1.53 - 1.20 (m, 36H, CH_2), 0.88 (t, *J* = 6.9 Hz, 6H, CH_3).

^{13}C-NMR (100 MHz, [D_6]Acetone, 25°C, TMS, ppm): δ = 155.6 (O*C*C), 155.3 (O*C*C), 143 (N*C*C), 142.7 (N*C*C), 125.6 (C*C*H), 125.5 (C*C*H), 116 (C*C*H), 116 (C*C*H), 68.8 (OCH_2), 67.9 (OCH_2), 34.5 ($BrCH_2$), 32.6 (CH_2), 30.5 (CH_2), 30.4 (CH_2), 28.8 (CH_2), 26.8 (CH_2), 23.3 (CH_2), 14.4 (CH_3).

IR (KBr, cm^{-1}): 2930 (s) (Csp^3-H), 2857 (s) (Csp^3-H), 1586 (w) (C-C_{Ar}), 1500 (vs) (C-C_{Ar}), 1237 (vs) (C-O *st*).

HRMS (ESI+) *m/z* calcd for $C_{46}H_{70}BrNO_3^+$: 763.4539 [*M*]$^+$; found: 763.4525 [M]$^+$.

Compounds 5(*R*) and 5(*S*).

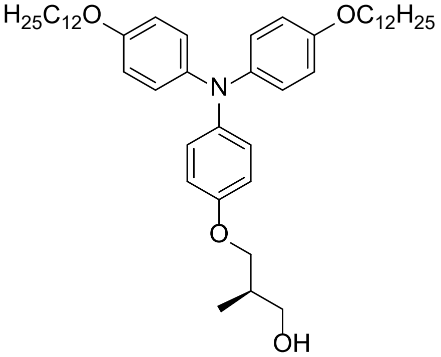

5(*R*)

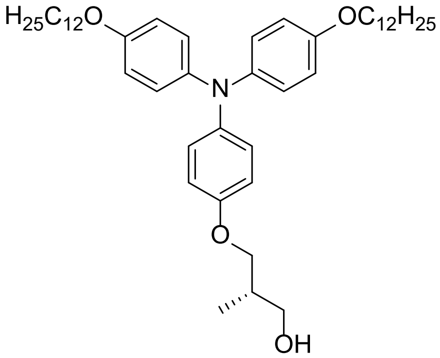

5(*S*)

Potassium carbonate (1.2 g, 2.1 mmol) and (*R* or *S*)-3-bromo-2-methylpropan-1-ol (0.3 g, 2.1 mmol) were added to a solution of compound **3** (1.1 g, 1.7 mmol) in butanone (20 mL) under an argon atmosphere. The resulted mixture was stirred overnight under reflux. After cooling at room temperature, the mixture was filtered through a celite®

pad, the solvent was removed under vacuum and the crude oil was purified by flash chromatography using hexane/ethyl acetate (95:5). Both products were obtained as colorless oils. Compound **5(*R*)** yield: 78%. Compound **5(*S*)** yield: 77%.

^{1}H-NMR (400 MHz, [D$_6$]Acetone, 25°C, TMS, ppm): δ = 6.94 - 6.88 (m, 6H, ArH), 6.87 - 6.80 (m, 6H, ArH), 3.97 (dd, *J* = 9.2 Hz, 1H, OCH), 3.95 (t, *J* = 6.8 Hz, 4H, OCH$_2$), 3.83 (dd, *J* = 9.2 Hz, 1H, OCH), 3.68 - 3.63 (m, 1H, OH), 3.62 - 3.53 (m, 2H, CH$_2$OH) 1.80 - 1.70 (m, 4H, CH$_2$), 1.53 - 1.20 (m, 36 H, CH$_2$), 1.03 (d, *J* = 6.9 Hz, 3H, CH$_3$), 0.89 (t, *J* = 6.8 Hz, 6H, CH$_3$).
^{13}C-NMR (100 MHz, [D$_6$]Acetone, 25°C, TMS, ppm): δ = 155.7 (O*C*C), 155.6 (O*C*C), 142.8 (N*C*C), 142.8 (N*C*C), 125.6 (C*C*H), 125.6 (C*C*H), 116.0 (C*C*H), 116.0 (C*C*H), 71.1(OCH$_2$), 68.8 (OCH$_2$), 37.1 (CH$_2$), 35.4 (CH$_2$), 31.9 (CH$_2$), 29.6 (CH$_2$), 29.4 (CH$_2$), 26.1 (CH$_2$), 22.7 (CH$_2$), 14.4 (CH$_3$), 14.3 (CH$_3$).
IR (KBr, cm^{-1}): 3421 (br) (OH), 2925 (vs) (Csp^3-H), 2854 (vs) (Csp^3-H), 1586 (w) (C-C$_{Ar}$), 1504 (vs) (C-C$_{Ar}$), 1237 (vs) (C-O *st*).
HRMS (ESI+) *m/z* calcd for $C_{46}H_{71}NO_4^+$: 701.5383 [*M*]$^+$; found: (***R***): 701.5388 [*M*]$^+$; (***S***): 701.5359 [*M*]$^+$.

Compounds 6(*S*) and 6(*R*).

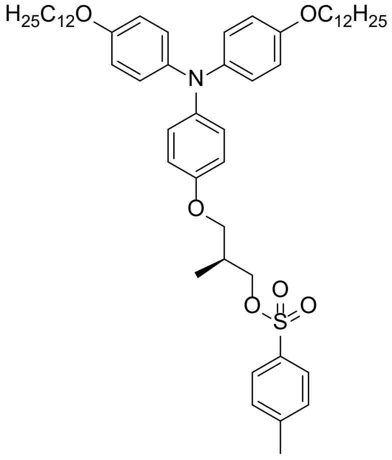

6(*S*)

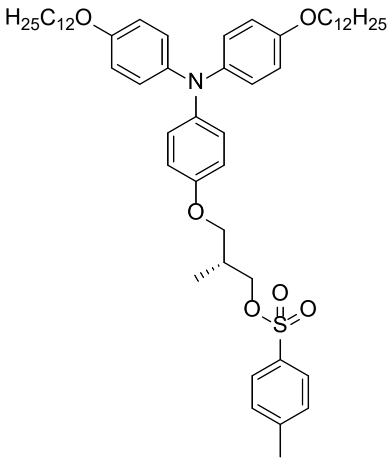

6(*R*)

p-Toluenesulfonyl chloride (0.5 g, 2.6 mmol) was added to a solution of compound **5(*R*)** or **5(*S*)** (0.74 g, 1.05 mmol) and triethylamine (0.21 g, 2.1 mmol) in dry dichloromethane under an argon atmosphere. The resulted mixture was stirred overnight at room temperature in the darkness to avoid TPA oxidation. After this time, the solvent was removed under reduced pressure and the crude oil was purified by flash chromatography starting with hexane/EtOAc (97.5:2.5) as initial solvent and finishing with hexane/ethyl acetate (95:5). Both products were obtained as colorless oils. Compound **6(*S*)** yield: 77%. Compound **6(*R*)** yield: 78%.

^{1}H-NMR (400 MHz, [D$_6$]Acetone, 25°C, TMS, ppm): δ = 7.81 - 7.86 (m, 2H, ArH,), 7.45 - 7.38 (m, 2H, ArH,), 6.95 - 6.81 (m, 10H, ArH), 6.76 - 6.70 (m, 2H, ArH), 4.17 - 4.06 (m, 2H, OCH$_2$), 3.95 (t, *J* = 6.5 Hz, 4H, OCH$_2$), 3.86 - 3.76 (m, 2H, OCH$_2$), 2.42 (s, 3H, CH$_3$ OTs), 1.80 - 1.70 (m, 4H, CH$_2$), 1.53 - 1.20 (m, 36 H, CH$_2$), 1.03 (d, *J* = 6.9 Hz, 3H, CH$_3$), 0.89 (t, *J* = 6.8 Hz, 6H, CH$_3$).

^{13}C-NMR (100 MHz, [D$_6$]Acetone, 25°C, TMS, ppm): δ = 155.7 (O*C*C), 155.0 (O*C*C), 145.8 (S*C*C), 143.1 (N*C*C), 142.7 (N*C*C), 130.9 (*C*CH$_3$), 128.7 (C*C*H), 125.7 (C*C*H), 125.3 (C*C*H), 116.0 (C*C*H), 72.6 (OCH$_2$), 69.4 (OCH$_2$), 68.8 (OCH$_2$), 34.2 (CH$_2$), 32.7 (CH$_2$), 26.9 (CH$_2$), 23.4 (CH$_2$), 21.7 (CH$_2$), 14.4 (CH$_3$), 13.6 (CH$_3$).
IR (KBr, cm^{-1}): 2926 (vs) (Csp^3-H), 2854 (vs) (Csp^3-H), 1599 (w) (C-C$_{Ar}$), 1504 (vs) (C-C$_{Ar}$), 1237 (vs) (C-O *st*).
HRMS (ESI+) *m/z* calcd for $C_{53}H_{78}NO_6S^+$: 856.5550 [*M*+H]$^+$; found:(***R***): 856.5509 [*M*+H]$^+$; (**S**): 856.5527 [*M*+H]$^+$.

General procedure for N-9 alkylation of adenine

NaH (0.03 g, 1.15 mmol) was added to a suspension of adenine (0.13 g, 1 mmol) in dry dimethylformamide under an argon atmosphere. The mixture was stirred at room temperature for one hour, until no more H_2 bubbling was observed. After this time, the corresponding bromide (**4**) or tosylate (**6(*R*) and 6(*S*)**) TPA derivative (1.3 mmol) was added, and the reaction mixture was stirred overnight at room temperature. Then, the solvent was evaporated under reduced pressure and the resulting crude was suspended in 50 mL of dichloromethane and filtered. The obtained solution was evaporated under reduced pressure and the residue purified by flash chromatography starting with dichloromethane as initial solvent and gradually increasing the polarity to dichloromethane /methanol 98:2.

Aden-C$_4$TPA

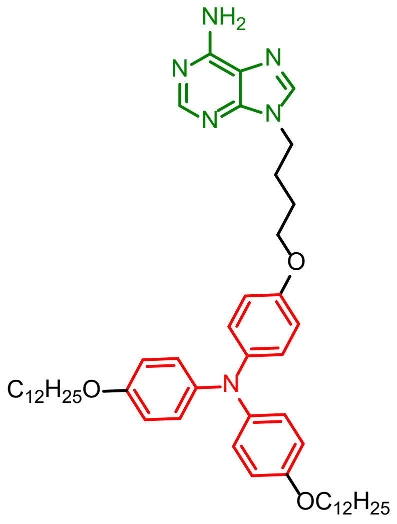

The product was recrystallized from ethanol obtaining a withe powder. Yield: 46%.

^{1}H-NMR (400 MHz, CDCl$_3$, 25°C, TMS, ppm): δ = 8.36 (s, 1H, ArH), 7.84 (s, 1H, ArH), 6.97 - 6.90 (m, 6H, ArH), 6.79 - 6.71 (m, 6H, ArH), 5.54 (s, 2H, NH$_2$), 4.30 (t, *J* = 7.2 Hz, 2H, NCH$_2$), 3.96 (t, *J* = 6.0 Hz, 2H, OCH$_2$), 3.90 (t, *J* = 6.6 Hz, 4H, OCH$_2$), 2.16 - 2.06 (m, 2H, CH$_2$), 1.85 - 1.70 (m, 6H, CH$_2$), 1.48 - 1.17 (m, 36 H, CH$_2$), 0.88 (t, *J* = 6.8 Hz, 6H, CH$_3$).
^{13}C-NMR (100 MHz, CDCl$_3$, 25°C, TMS, ppm): δ = 155.5 (C*C*NH$_2$), 154.7 (O*C*C), 154 (O*C*C), 153.1 (N*C*H), 150.4 (N*C*C), 142.5 (N*C*C), 141.9 (N*C*C), 140.7 (N*C*H), 125.1 (C*C*H), 124.7 (C*C*H), 119.9 (N*C*C), 115.3 (C*C*H), 115.2 (C*C*H), 68.5 (OCH$_2$), 67.6 (OCH$_2$), 43.8 (NCH$_2$), 32.1 (CH$_2$), 29.8 (CH$_2$), 29.8 (CH$_2$), 29.8 (CH$_2$), 29.7 (CH$_2$), 29.6 (CH$_2$), 29.5 (CH$_2$), 29.5 (CH$_2$), 27.3 (CH$_2$), 26.6 (CH$_2$), 26.2 (CH$_2$), 22.8 (CH$_2$), 14.3 (CH$_3$).
IR (KBr, cm^{-1}): 3287 (br) (N-H *st*), 3124 (br) (N-H *st*), 2923 (vs) (Csp^3-H), 2852 (vs) (Csp^3-H), 1680 (s) (N-H δ), 1602 (s) (C-N *st*), 1572 (w) (C-C$_{Ar}$), 1504 (vs) (C-C$_{Ar}$), 1237 (vs) (C-O *st*).
HRMS (ESI+) *m/z* calcd for $C_{51}H_{74}N_6O_3^+$: 818.5822 [*M*]$^+$; found: 818.5812 [*M*]$^+$.

Aden-(*R*)C$_3$*TPA and **Aden-(*S*)C$_3$*TPA**

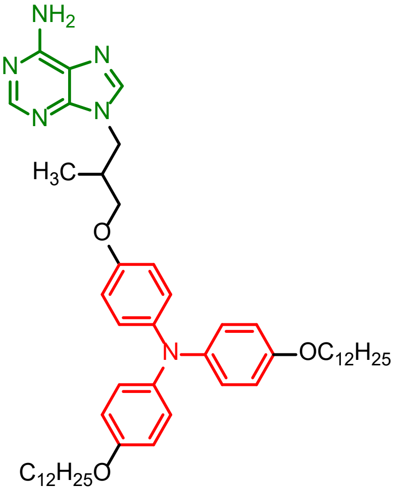

The products were recrystallized from methanol obtaining sticky colorless solids. Compound **Aden-(*R*)C$_3$*TPA** yield: 39%. Compound **Aden-(*S*)C$_3$*TPA** yield: 41%.

^{1}H-RMN (400 MHz, CDCl$_3$, 25°C, TMS, ppm): δ= 8.37 (s, 1H, ArH), 7.76 (s, 1H, ArH), 6.98 - 6.89 (m, 6H, ArH), 6.81 - 6.70 (m, 6H, ArH), 5.56 (s, 2H, NH$_2$), 4.37 (dd, *J* = 14, 7.2 Hz, 1H, NCH$_2$), 4.26 (dd, *J* = 14, 7.2 Hz, 1H, NCH$_2$), 3.91 (t, *J* = 6.6 Hz, 4H, OCH$_2$), 3.82 - 3.72 (m, 2H, OCH$_2$), 2.68 - 2.55 (m, 1H, CH), 1.81 - 1.70 (m, 4H, CH$_2$), 1.50 - 1.20 (m, 36H, CH$_2$), 1.10 (d, *J* = 6.9 Hz, 3H, CH$_3$), 0.88 (t, *J* = 6.9 Hz, 6H, CH$_3$).
^{13}C-RMN (100 MHz, CDCl$_3$, 25°C, TMS, ppm): δ= 155.5 (C*C*NH$_2$), 154.7 (O*C*C), 153.7 (O*C*C), 153.2 (N*C*H), 150.6 (N*C*C), 142.7 (N*C*C), 141.8 (N*C*C), 141.4 (N*C*H), 125.1 (C*C*H), 124.5 (C*C*H), 119.7 (N*C*C), 115.3 (C*C*H), 115.1 (C*C*H), 69.7 (OCH$_2$), 68.4 (OCH$_2$), 46.6 (NCH$_2$), 34.2 (CH), 32 (CH$_2$), 29.8 (CH$_2$), 29.7 (CH$_2$), 29.6 (CH$_2$), 29.5 (CH$_2$), 29.4 (CH$_2$), 26.2 (CH$_2$), 22.8 (CH$_2$), 15 (CH$_3$), 14.3 (CH$_3$).
IR (KBr, cm^{-1}): 3307 (br) (N-H *st*), 3149 (br) (N-H *st*), 2933 (vs) (Csp^3-H), 2855 (vs) (Csp^3-H), 1644 (s) (N-H δ), 1601 (s) (C-N *st*), 1575 (w) (C-C$_{Ar}$), 1504 (vs) (C-C$_{Ar}$), 1236 (vs) (C-O *st*).
HRMS (ESI+) *m/z* calcd for $C_{51}H_{75}N_6O_3^+$: 819.5901 [*M*+H]$^+$; found: (*R*): 819.5881 [*M*+H]$^+$, (S): 819.5871 [*M*+H]$^+$.

^{1}H NMR and ^{13}C NMR spectra

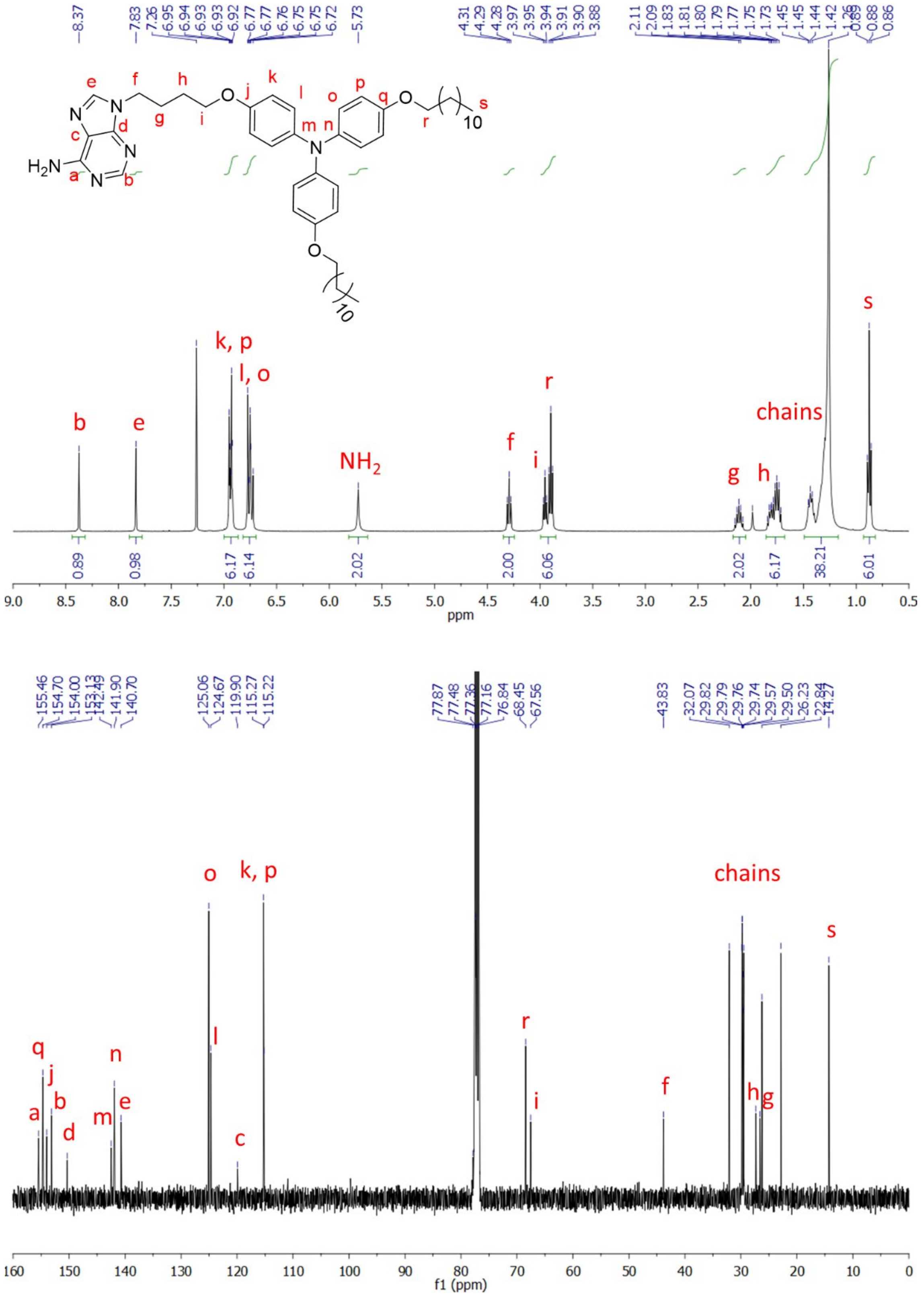

Figure S1. ^{1}H-NMR and ^{13}C-NMR spectra of compound **Aden-C$_4$TPA** in $CDCl_3$.

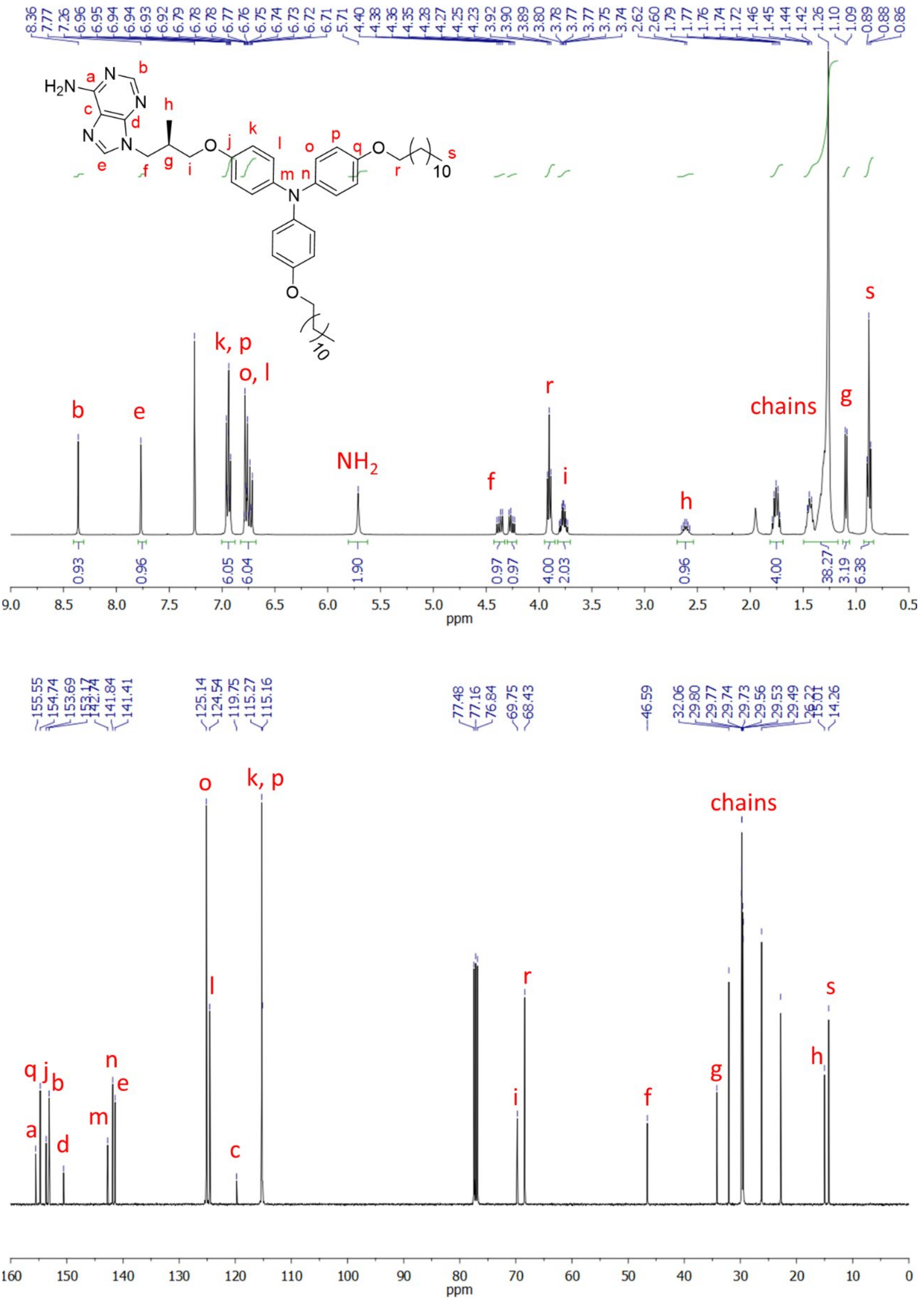

Figure S2. ^{1}H-NMR and ^{13}C-NMR spectra of compound **Aden-(*R*)C₃*TPA** in CDCl₃.

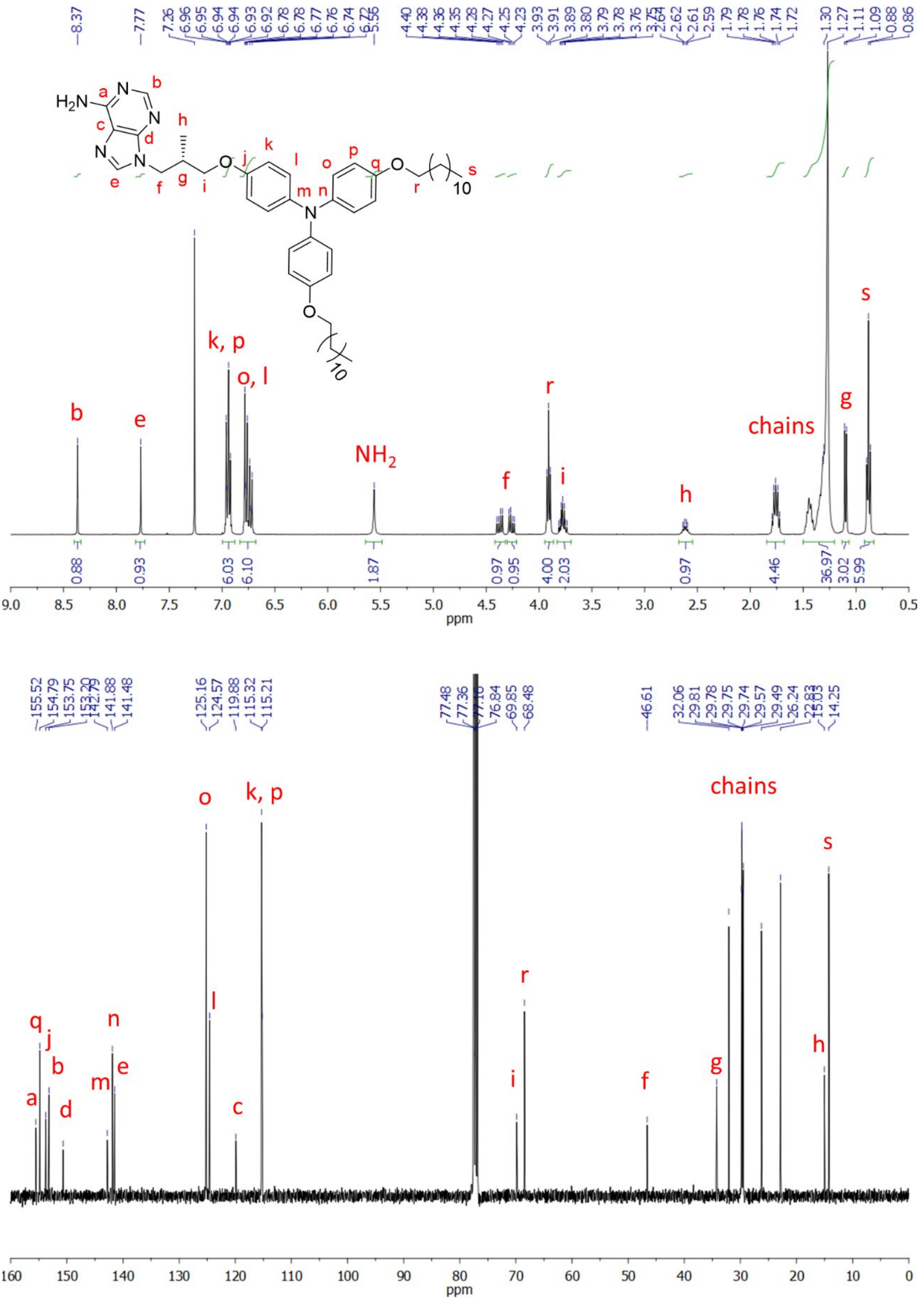

Figure S3. ^{1}H-NMR and ^{13}C-NMR spectra of compound **Aden-(*S*)C$_3$*TPA** in $CDCl_3$.

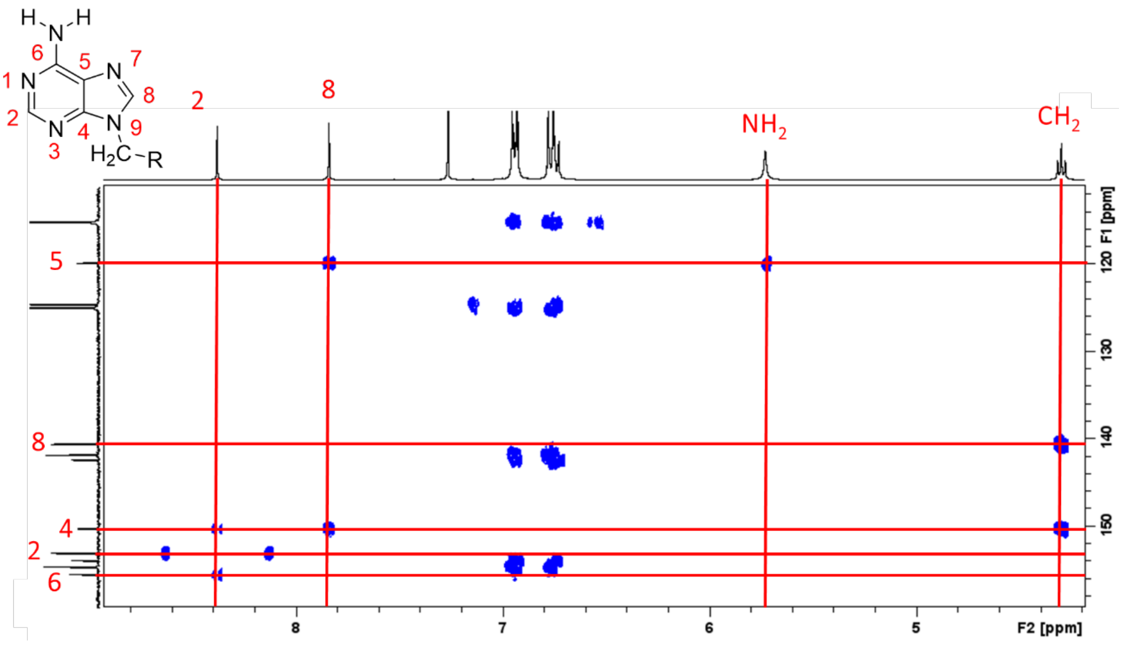

Figure S4. HMBC spectra of compound **Aden-C$_4$TPA** in $CDCl_3$.

Thermal properties. DSC thermograms

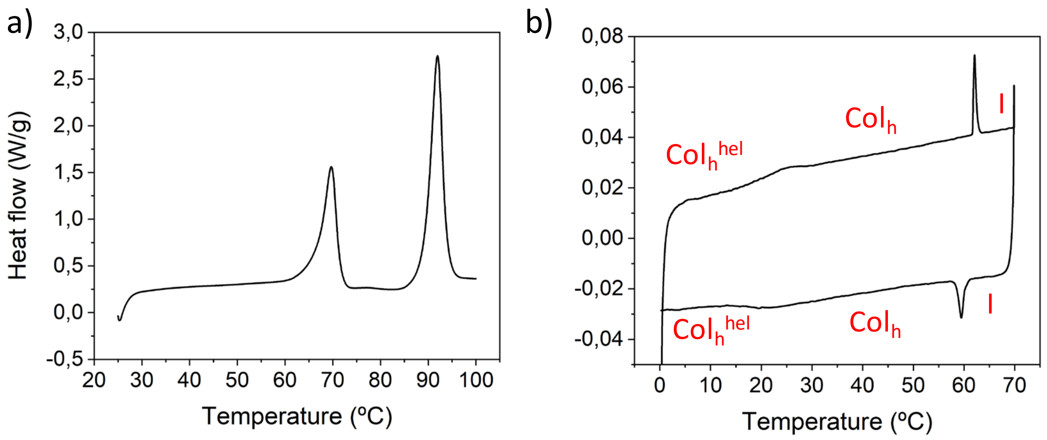

Figure S5. DSC thermograms of compound **Aden-C$_4$TPA** (a) first heating cycle of the as-prepared sample recorded at a rate of 10 °C/min, (b) first cooling cycle and second heating cycle recorded at a rate of 1 °C/min.

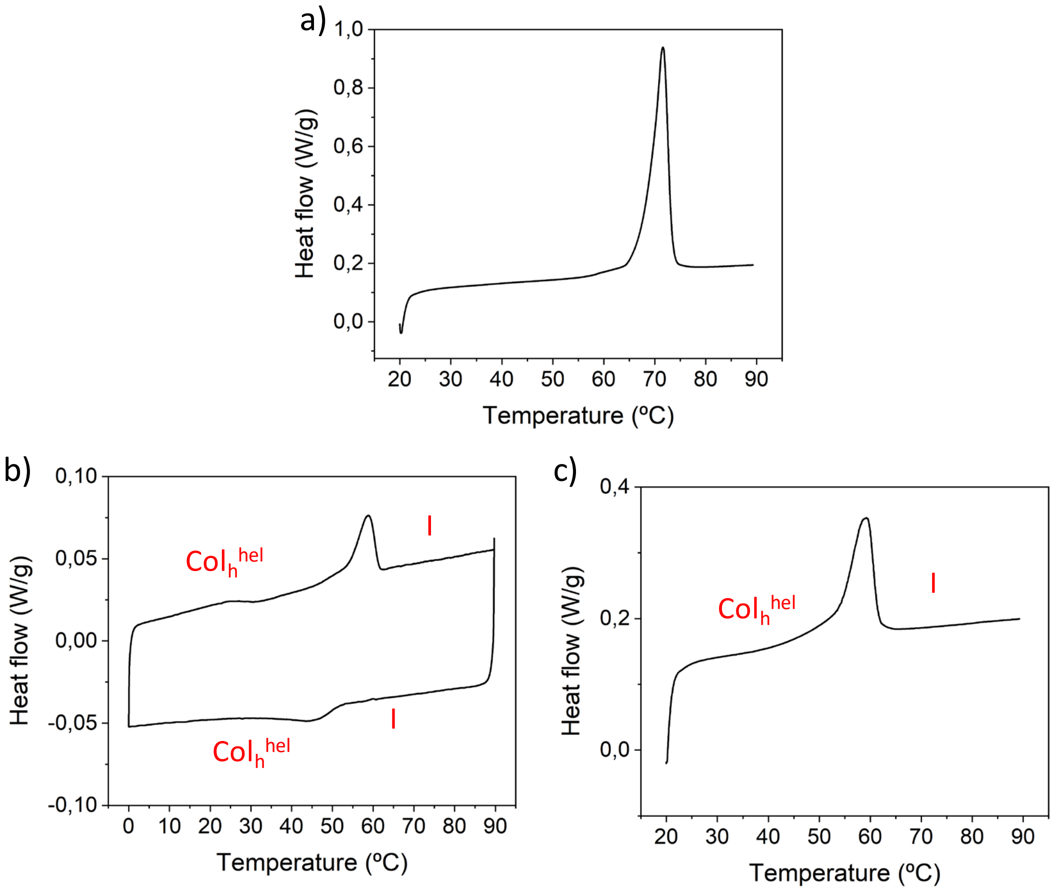

Figure S6. DSC thermograms of compound **Aden-(*R*)C$_3$*TPA** (a) first heating cycle of the as-prepared sample recorded at a rate of 10 °C/min, (b) first cooling cycle and second heating cycle recorded at a rate of 1 °C/min and (c) third heating cycle recorded at a rate of 5 °C/min after 24h at room temperature.

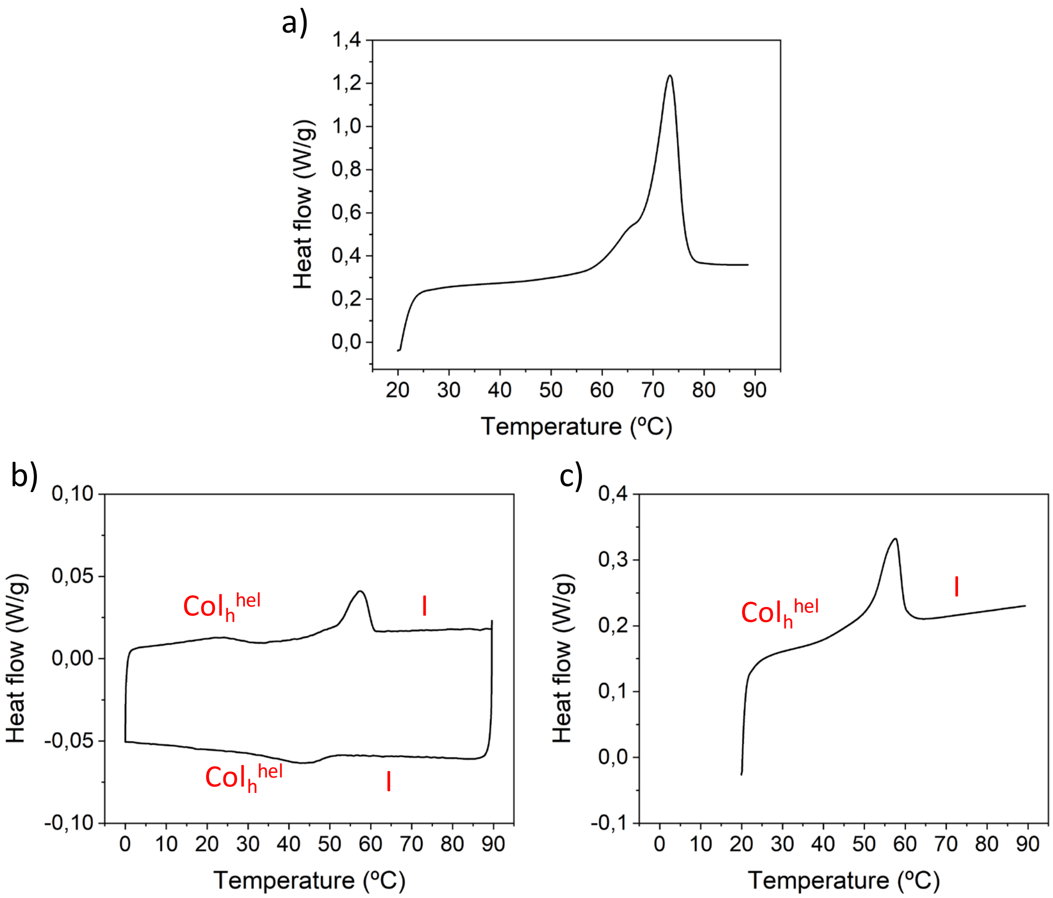

Figure S7. DSC thermograms of compound **Aden-(*S*)C$_3$*TPA** (a) first heating cycle of the as-prepared sample recorded at a rate of 10 °C/min, (b) first cooling cycle and second heating cycle recorded at a rate of 1 °C/min and (c) third heating cycle recorded at a rate of 5 °C/min after 24h at room temperature.

XRD studies

Aden-C_4TPA at r.t.

hkl	d observed (Å)
100	45.1
110	26.0
120	17.1
001	7.7
002	3.8

a = 52.1 Å; λ = 81.9 Å $\Longrightarrow$ 10.6 units of 7.7 Å $\Longrightarrow$ α = 5.6°

Aden-C_4TPA at 45 ºC

hkl	d observed (Å)
100	41.1
110	24.0

a = 47.4 Å

Aden-(*S*)C_3*TPA

hkl	d observed (Å)
100	44.2
110	25.8
200	22.5
120	17.0
001	7.7
002	3.8

a = 51.0 Å; λ = 76.3 Å $\Longrightarrow$ 9.9 units of 7.7 Å $\Longrightarrow$ α = 6.0°

<u>**Calculation of Z**</u>

The number of molecules per unit cell (Z) in the Col_h mesophase was estimated with the density equation $\rho = (M \cdot Z)/(N_A . V)$, where the volume of the unit cell can be calculated with the equation $V = a^2 \cdot \surd 3/2 \cdot c$ and therefore, the first equation can be restructured as $Z = (\rho \cdot a^2 \cdot \surd 3/2 \cdot c \cdot N_A)/M$.

Aden-C_4TPA

Z = ($0.98\ g \cdot cm^{-3}$ x $(52.1\ Å)^2$x √3/2 x 3.8 Å x $6.022 \cdot 10^{23}\ mol^{-1}$) / (819.13 $g \cdot mol^{-1}$ x 10^{24} $Å^3 \cdot cm^{-3}$) = 6.4 molecules

Aden-C_3*TPA

Z = ($0.93\ g \cdot cm^{-3}$ x $(51\ Å)^2$x √3/2 x 3.8 Å x $6.022 \cdot 10^{23}\ mol^{-1}$) / (819.13 $g \cdot mol^{-1}$ x 10^{24} $Å^3 \cdot cm^{-3}$) = 5.9 molecules

Adenine hexamer optimization

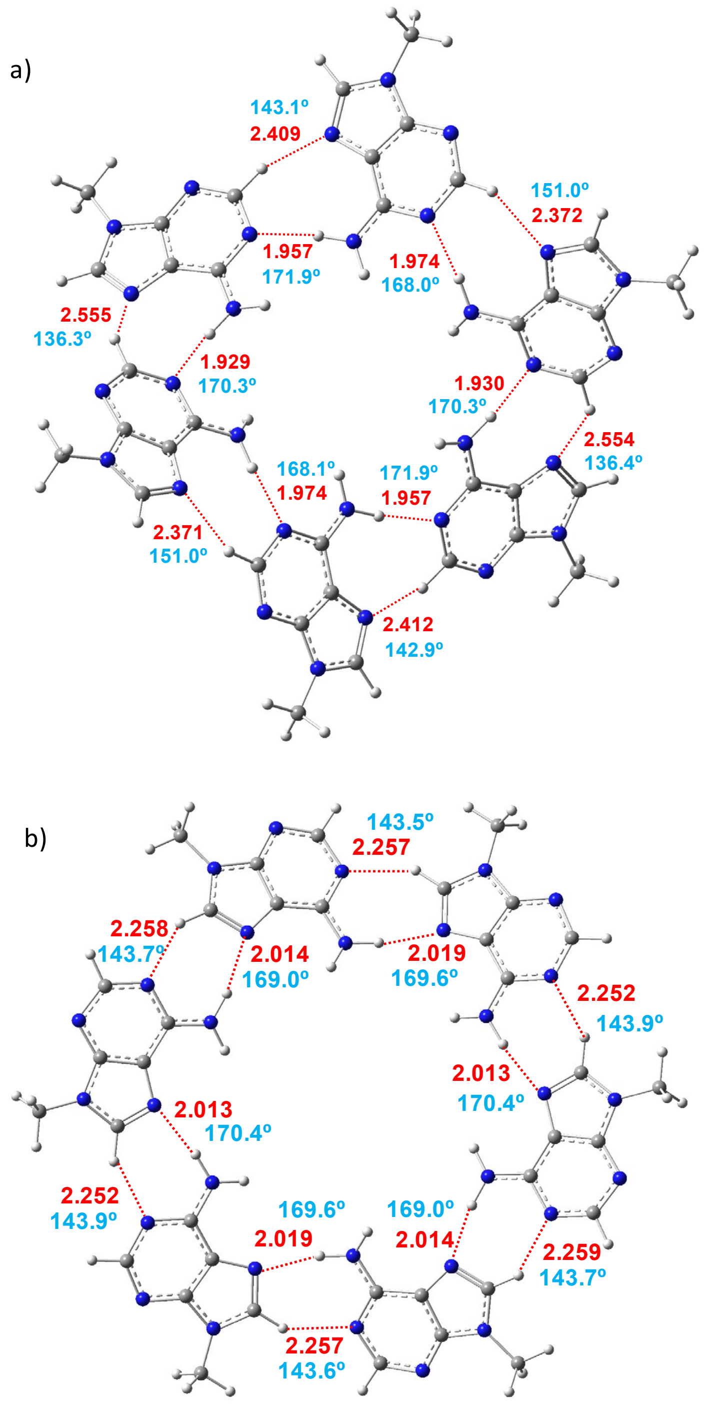

Figure S8. Optimized geometries of (a) A_6-N1 and (b) A_6-N7 including H-bond distances (Å, in red) and angles (in blue) calculated at the ωB97xD/6-31G* level of theory.

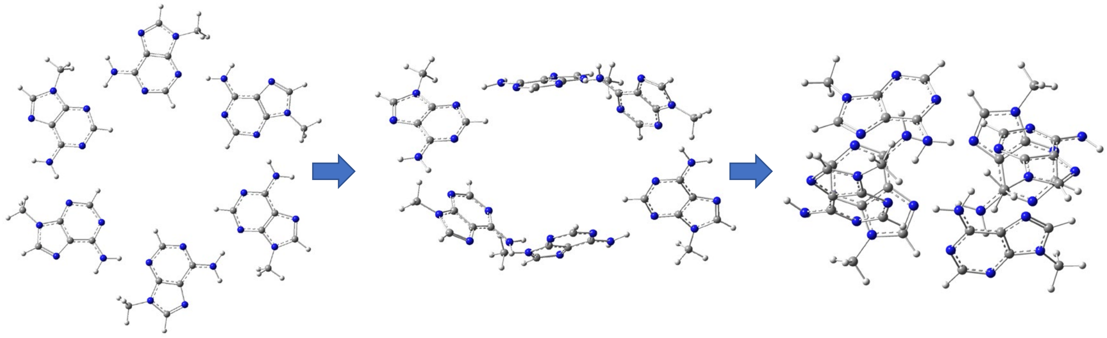

Figure S9. Evolution of the geometry optimization of A_6-N3 at the ωB97xD/6-31G* level of theory. The image shows how the cycle folds from the start point to the end point of the optimization.

Table S1. Energies (in Hartrees) and relative energies (ΔE in Kcal/mol) for the optimized structures of A_6-N1 (R = C_4TPAOCH$_3$), A_6-N7 (R = C_4TPAOCH$_3$), A_6-N1, A_6-N3 and A_6-N7 calculated at the ωB97xD/6-31G* level of theory.

Compound	Energy	ΔE
A_6-N1 (R = C_4TPAOCH$_3$)	-10060.846856	16.5
A_6-N7 (R = C_4TPAOCH$_3$)	-10060.873161	0.0
A_6-N1	-3038.902374	6.9
A_6-N7	-3038.913446	0.0
A_6-N3	-3038.958031	-

Model for the Col_h^{hel} phase

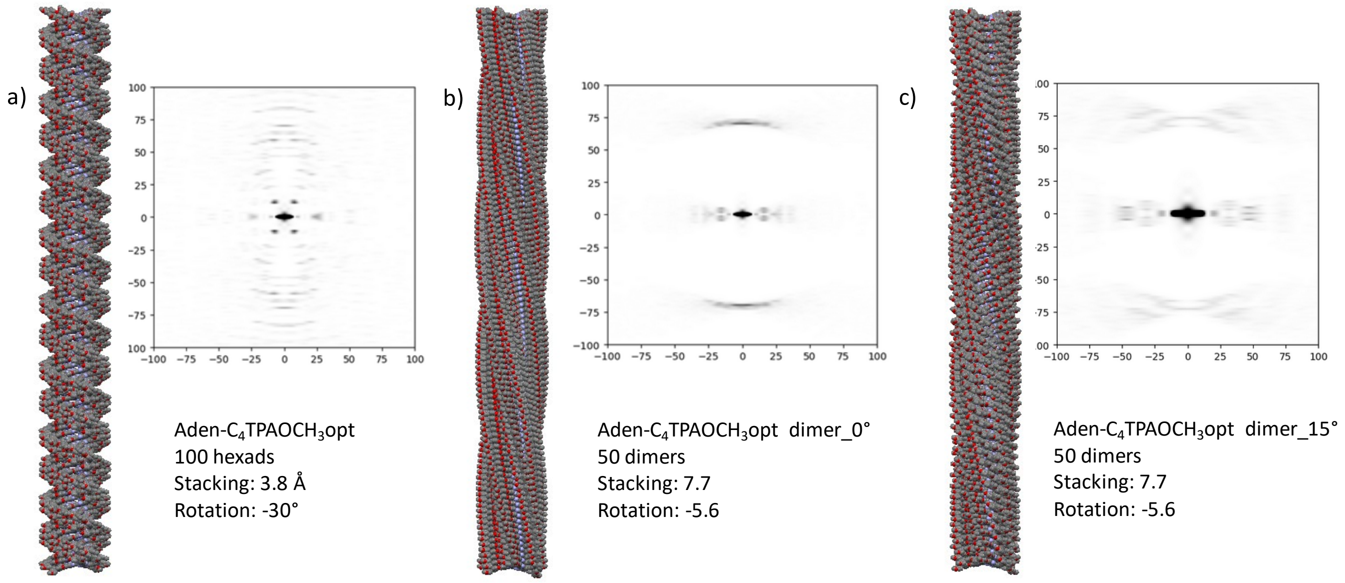

Figure S10. Simulated XRD pattern for different stacking models used to support the helical stacking model proposed in Figure 4 and S11. Aden-$C_4TPAOCH_3$opt stands for the optimized A_6-N7 hexamer. (a) 100 hexamers rotated -30°. (b) 50 dimers rotated -5.6°, each dimer made of two stacked hexameric rosettes mutually rotated by 0°. (c) 50 dimers rotated -5.6°, each dimer made of two stacked hexameric rosettes mutually rotated by 15°.

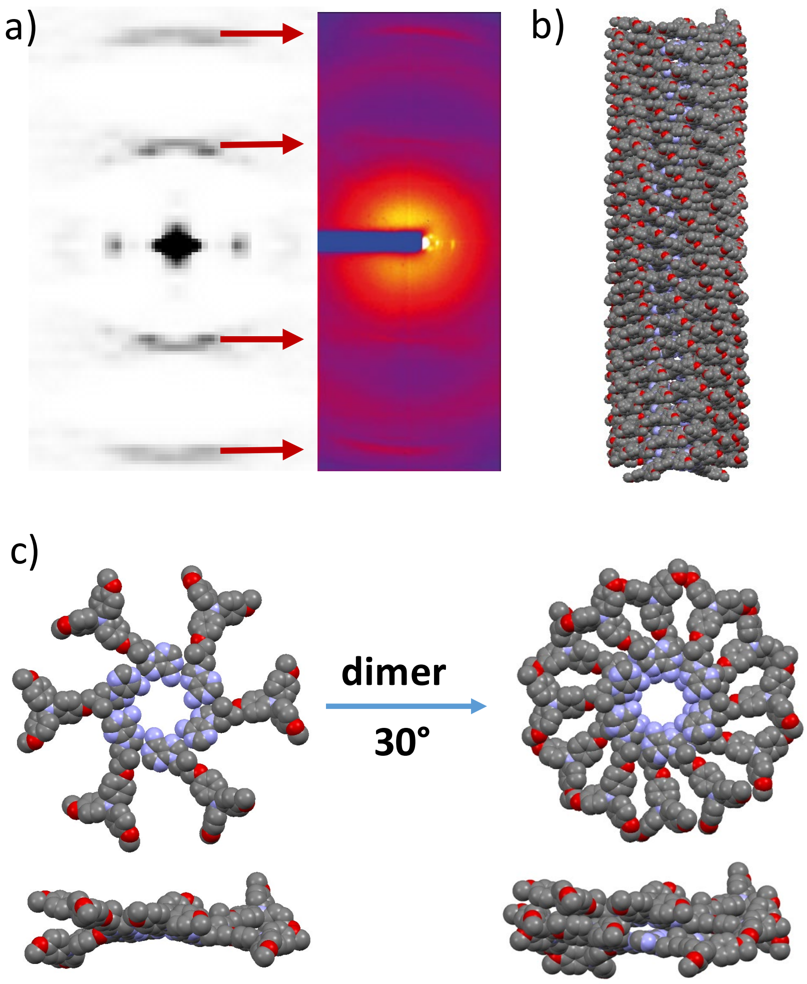

Figure S11. (a) Simulated XRD pattern for an idealized helical column of A_6-N7 (R = **(*R*)C$_3$*TPAOCH$_3$**) consisting on 20 dimers rotated -6° (left), and their comparison with the experimental XRD pattern of **Aden-(*R*)C$_3$*TPA** at r.t. (right). (b) Helical column obtained from the simulation. (c) Each dimer is made of two stacked hexameric rosettes mutually rotated by 30°.

Hydrogen bond studies by FTIR

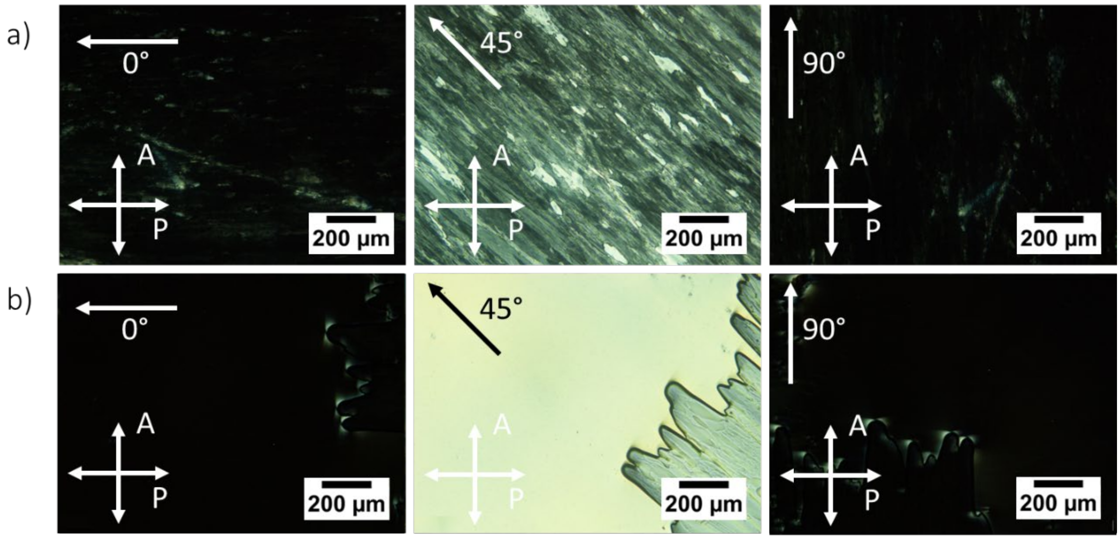

Figure S12. POM textures of partially aligned sample of compounds (a) **Aden-C_4TPA**, and (b) **Aden-(*R*)C_3*TPA** between KBr plates. The alignment direction is indicated with an arrow.

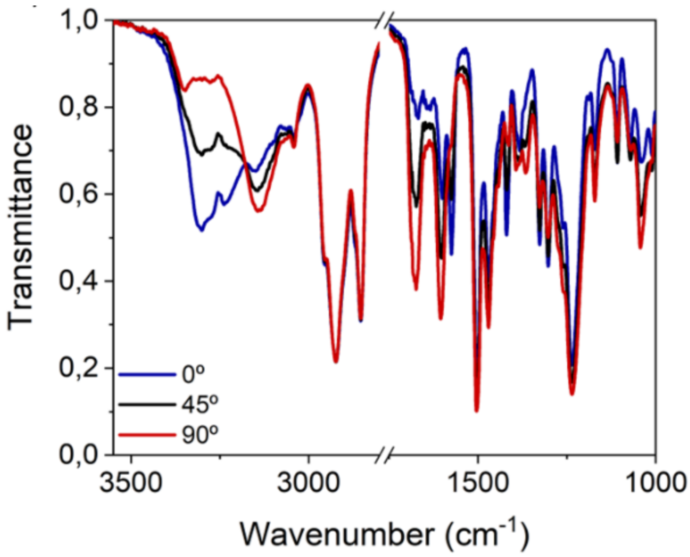

Figure S13. Polarized FTIR spectra of compound **Aden-(*R*)C_3*TPA** recorded at different angles.

a)

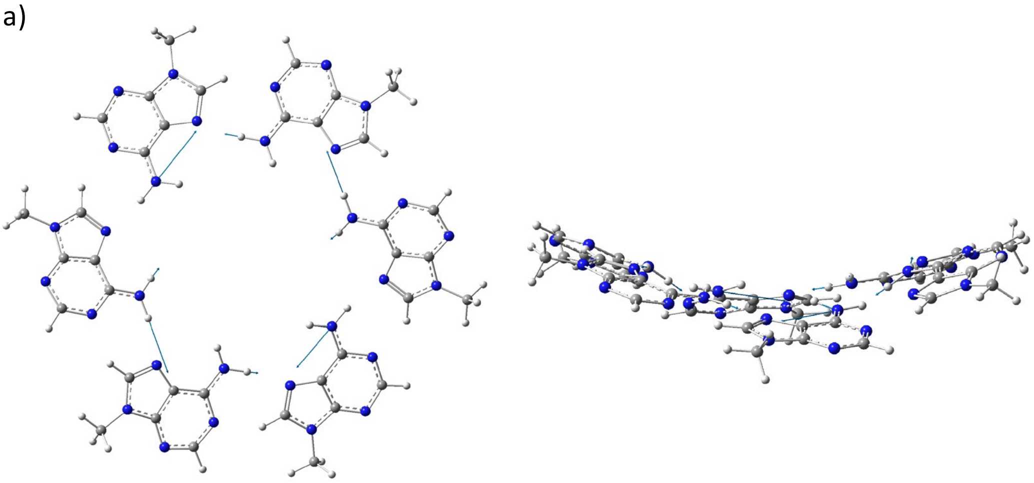

b)

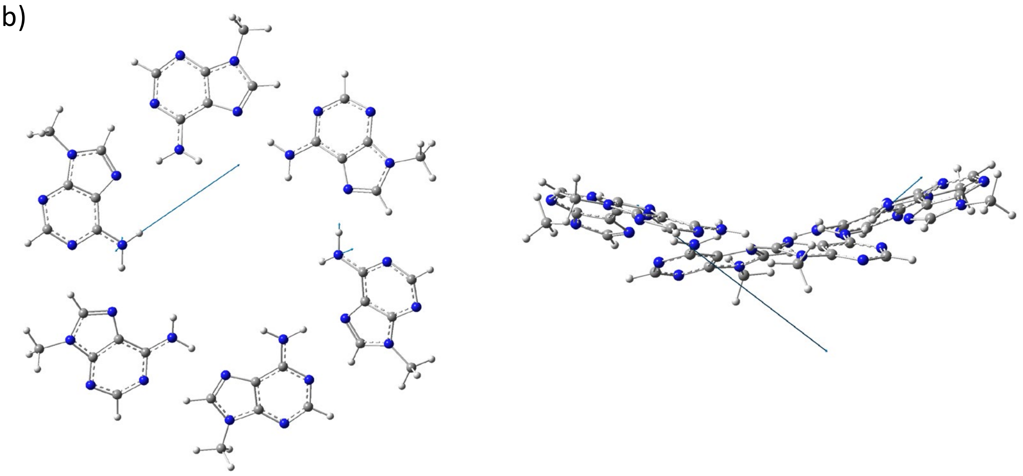

Figure S14. Representation of the atomic displacements predicted for the symmetric (a) and asymmetric (b) NH_2 *st* vibrational modes performed on the A_6-N7 hexamer optimized at the ωB97xD/6-31G* theory level.

Table S2. Theoretical and scaled vibrational modes (in cm^{-1}) for A_6-N7 calculated at the ωB97xD/6-31G* level of theory.

Assignment	Theoretical	Scaled*
	3737	3644
	3737	3644
Stretching NH asym	3735	3642
	3735	3641
	3725	3632
	3725	3632
	3463	3376
	3462	3375
Stretching NH sym	3459	3372
	3455	3369
	3451	3365
	3446	3360
	3240	3159
	3240	3159
Stretching CH (imidazol)	3238	3157
	3238	3157
	3236	3155
	3236	3155
	3206	3126
	3206	3126
Stretching CH (pyrimidine)	3205	3125
	3205	3125
	3204	3124
	3204	3124
	1736	1692
	1733	1690
	1732	1688
NH_2 bending	1731	1688
	1730	1687
	1730	1687

*Scale factor 0.975[2]

Chirooptical properties. CD spectra

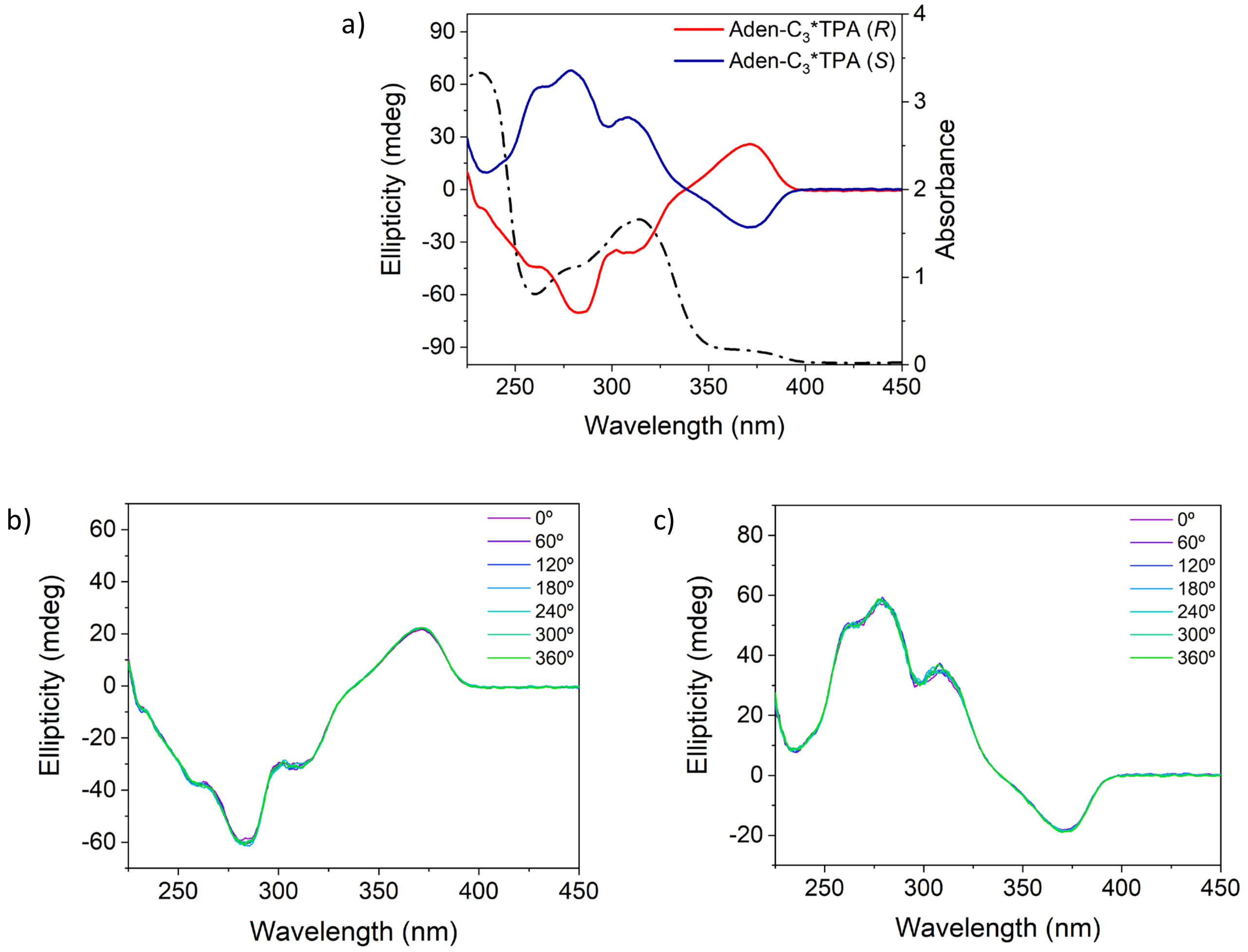

Figure S15. (a) CD spectra recorded for **Aden-(*R*)C₃*TPA** (red line) and **Aden-(*S*)C₃*TPA** (blue line), and absorption spectra (black dotted line) at r.t. CD spectra of compounds (b) **Aden-(*R*)C₃*TPA** and (c) **Aden-(*S*)C₃*TPA** in the mesophase recorded every 60° by rotating the sample around the measurement direction.

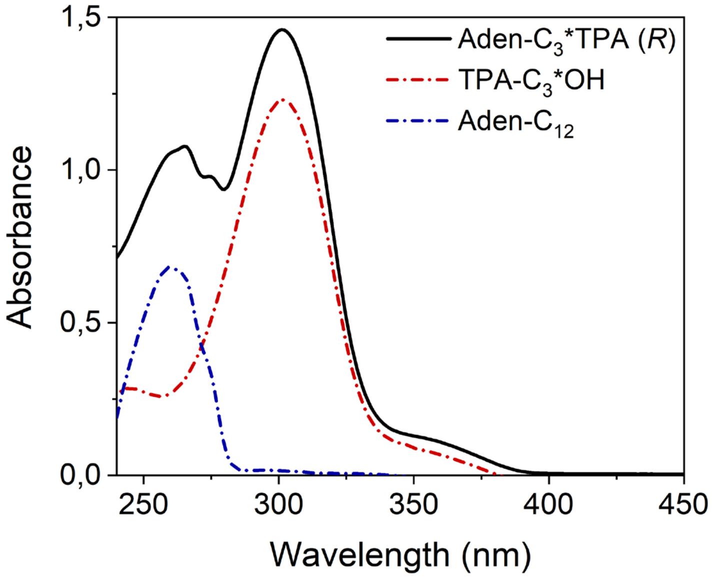

Figure S16. UV-vis spectrum of compounds **Aden-(*R*)C$_3$*TPA**, **5(*R*)** (**TPA-C$_3$*OH)** and 9-dodecyladenine (**Aden-C$_{12}$**) in $CDCl_3$ solution (10^{-5} M).

Electrochemical properties and energy levels

Table S3. Electrochemical data in solution.

Compound	E^{red} (V) *vs* Ag/AgCl	$E^{ox}_{1/2}$ (V) *vs* Ag/AgCl	E^{red} (V)[b] *vs* FOC	$E^{ox}_{1/2}$ (V)[b] *vs* FOC	HOMO (eV)[c]	LUMO (eV)[d]
**Aden-C_4TPA** [a]	-	0.53	-	0.11	-4.91	-
**Aden-C_3*TPA** [a]	-	0.52	-	0.10	-4.90	-

[a] In dichloromethane solution. [b]$E_{1/2}$ = 0.42 V *vs* Ag/AgCl. [c]E_{HOMO} = -e[$E^{ox}_{1/2}$ *vs* FOC + 4.8 V]. [d]E_{LUMO} = -e[E^{red} *vs* FOC + 4.8 V].

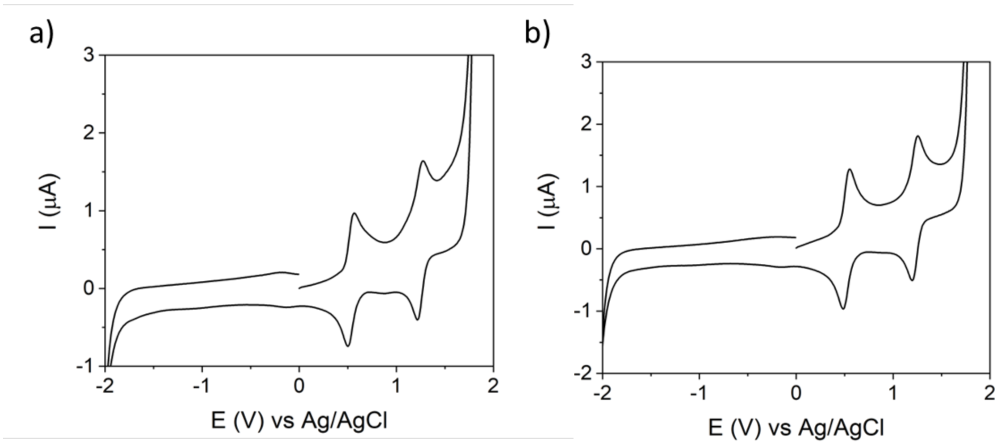

Figure S17. Cyclic voltammograms recorded for (a) **Aden-C_4TPA** and (b) **Aden-C_3*TPA** (***R*** or ***S***) in dichloromethane solution ($1{\cdot}10^{-4}$ M) and NBu_4PF_6 (0.1 M).

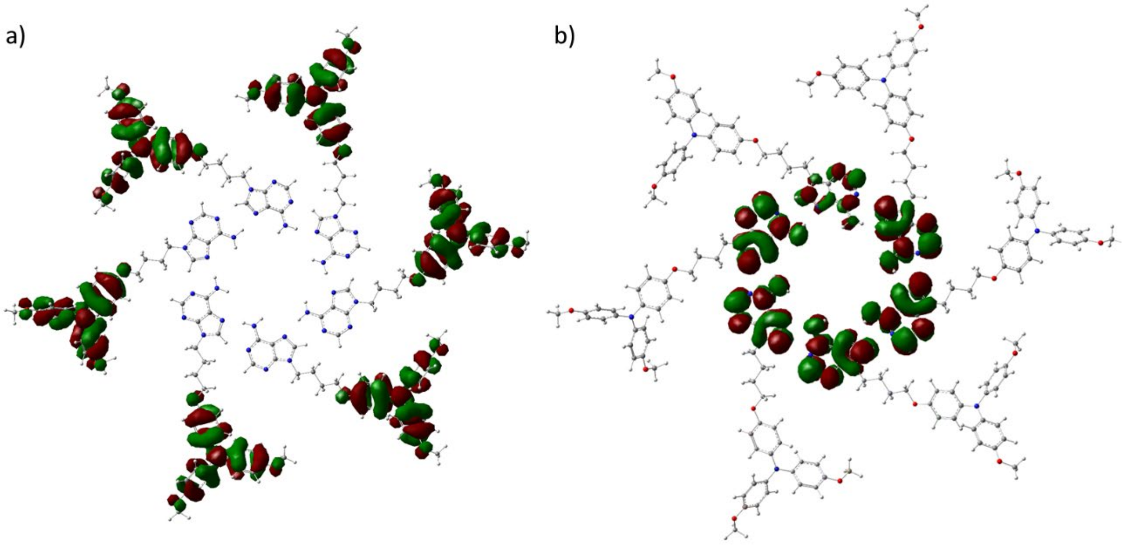

Figure S18. Molecular orbital representation (isocontour plots 0.02 a.u.) of a) HOMO,HOMO-1,HOMO-2, HOMO-3, HOMO-4, HOMO-5 and b) LUMO, LUMO+1, LUMO+2, LUMO+3, LUMO+4, LUMO+5 determined for A_6-N7 (R=C4TPAOCH3) calculated at the ωB97xD/6-31G* level of theory.

Table S4. Molecular orbital energy levels (in eV) for A_6-N7 (R = $C_4TPAOCH_3$) and A_6-N1 (R = $C_4TPAOCH_3$) calculated at the ωB97xD/6-31G* level of theory.

	A_6-N7 (R=$C_4TPAOCH_3$)	*A_6-N1 (R=$C_4TPAOCH_3$)*
LUMO+5	*1,46*	*1,56*
LUMO+4	*1,41*	*1,55*
LUMO+3	*1,41*	*1,55*
LUMO+2	*1,39*	*1,55*
LUMO+1	*1,33*	*1,53*
LUMO	*1,31*	*1,50*
HOMO	*-6,26*	*-6,29*
HOMO-1	*-6,27*	*-6,29*
HOMO-2	*-6,27*	*-6,30*
HOMO-3	*-6,28*	*-6,30*
HOMO-4	*-6,30*	*-6,30*
HOMO-5	*-6,32*	*-6,32*

Charge mobility measurements

The charge mobility of the three materials was measured by the Space-Charge Limited Current (SCLC) method in solution processed samples. It entails the acquisition of the electric current flowing through the material, placed between two electrodes, as a function of the applied voltage. For low applied voltages, the current follows Ohm's law, while at higher voltages, if it is limited by a space-charge field, the current dependence on voltage is quadratic, following the Mott-Gurney law, in which the effect of the traps is neglected:

$$J = \frac{9}{8}\varepsilon\varepsilon_0\mu\frac{V^2}{d^3}$$

Where *J* is the current density, ε is the relative dielectric constant of the material, ε_0 is the dielectric constant of the vacuum, *V* is the applied voltage and *d* is the sample thickness.

To obtain a good charge injection between the electrode and the material, the contact between both should be ohmic, i.e. the difference between the HOMO (in the case of p-conductors) or the LUMO (in the case of n-conductors) level energy of the material and the work function of the injecting electrode must not be higher than 0.3-0.4 eV. Since in the case of these materials the energy of HOMO level is 5.1 eV, Au electrodes (work function 5.0-5.1 eV) were used to obtain ohmic contact in the hole measurements cells.

Hole mobility devices (Figure S18) were prepared by spin-coating 110 µL of a solution of the material in $CHCl_3$ (350 nm thickness, 30 mg/mL at 1500rpm during 1 min) onto a glass covered by 3 ITO stripes. Afterwards, 3 Au stripes (100 nm thickness) orthogonal to ITO stripes were deposited on top of the material layer by evaporation under vacuum. In this way, 9 independent 0.6 mm^2 areas between both electrodes are obtained. The samples were annealed 10 °C below the clearing point during one hour in order to favor the correct mesophase formation and orientation.

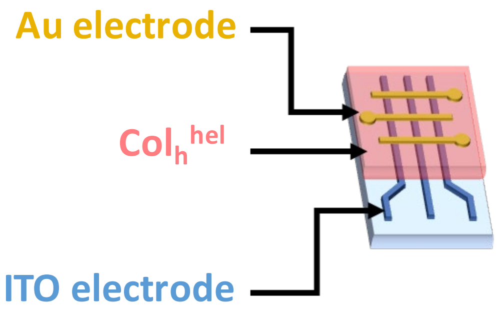

Figure S19. Schematic structure of the device prepared for SCLC measurements

I/V measurement was carried out using an electrometer, either a Keithley 6517A or a Keithley 2636B. An HP 4284A Precision LCR Meter was used to measure the capacity of the cells and obtain the dielectric constant.

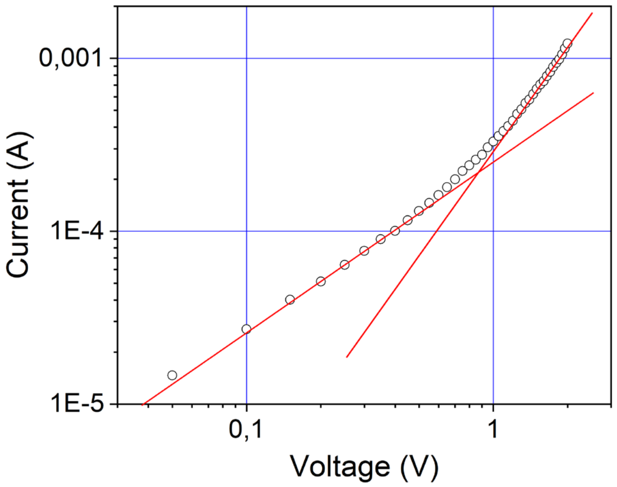

Figure S20. Typical *J-V* curve observed for compound **Aden-(*S*)C$_3$*TPA** at r.t. The straight lines show ideal linear and quadratic dependence of current on applied voltage

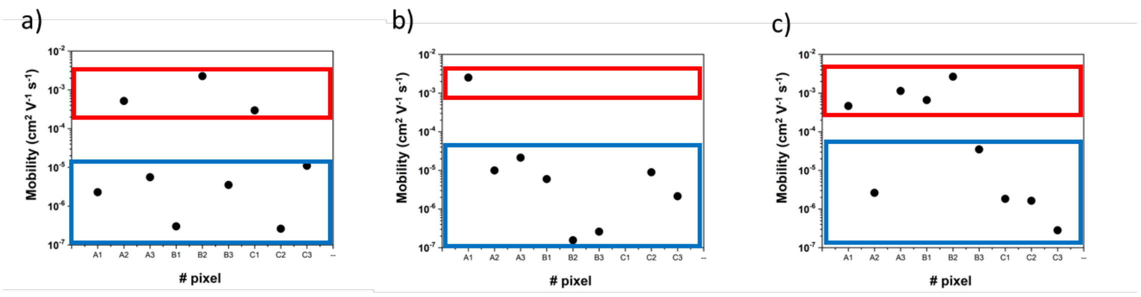

$1.0 \pm 1.0 \cdot 10^{-3}$ $cm^2 \cdot s^{-1} \cdot V^{-1}$

$3.8 \pm 4.0 \cdot 10^{-6}$ $cm^2 \cdot s^{-1} \cdot V^{-1}$

Aden-C_4TPA

$2.5 \pm 0.1 \cdot 10^{-3}$ $cm^2 \cdot s^{-1} \cdot V^{-1}$

$7.0 \pm 7.5 \cdot 10^{-6}$ $cm^2 \cdot s^{-1} \cdot V^{-1}$

Aden-(*R*)C_3*TPA

$1.2 \pm 1.0 \cdot 10^{-3}$ $cm^2 \cdot s^{-1} \cdot V^{-1}$

$0.7 \pm 1.4 \cdot 10^{-5}$ $cm^2 \cdot s^{-1} \cdot V^{-1}$

Aden-(*S*)C_3*TPA

Figure S21. Hole mobility measurements of compounds (a) **Aden-C_4TPA**, (b) **Aden-(*R*)C_3*TPA** and (c) **Aden-(*S*)C_3*TPA** in different pixels (labelled A1-3, B1-3 and C1-3).

References

1 B. Feringán, R. Termine, A. Golemme, J. M. Granadino-Roldán, A. Navarro, R. Giménez and T. Sierra, *J. Mater. Chem. C*, 2021, **9**, 1972-1982.

2 M. J. Frisch, G. W. Trucks, H. B. Schlegel, G. E. Scuseria, M. A. Robb, J. R. Cheeseman, G. Scalmani, V. Barone, B. Mennucci, G. A. Petersson, H. Nakatsuji, M. Caricato, X. Li, H. P. Hratchian, A. F. Izmaylov, J. Bloino, G. Zheng, J. L. Sonnenberg, M. Hada, M. Ehara, K. Toyota, R. Fukuda, J. Hasegawa, M. Ishida, T. Nakajima, Y. Honda, O. Kitao, H. Nakai, T. Vreven, J. A. Montgomery, Jr., J. E. Peralta, F. Ogliaro, M. Bearpark, J. J. Heyd, E. Brothers, K. N. Kudin, V. N. Staroverov, R. Kobayashi, J. Normand, K. Raghavachari, A. Rendell, J. C. Burant, S. S. Iyengar, J. Tomasi, M. Cossi, N. Rega, J. M. Millam, M. Klene, J. E. Knox, J. B. Cross, V. Bakken, C. Adamo, J. Jaramillo, R. Gomperts, R. E. Stratmann, O. Yazyev, A. J. Austin, R. Cammi, C. Pomelli, J. W. Ochterski, R. L. Martin, K. Morokuma, V. G. Zakrzewski, G. A. Voth, P. Salvador, J. J. Dannenberg, S. Dapprich, A. D. Daniels, Ö. Farkas, J. B. Foresman, J. V. Ortiz, J. Cioslowski, and D. J. Fox, Gaussian 16, Revision A.03. Gaussian, Inc., Wallingford CT, 2016.
